# Enteral Nutrition Versus a Combination of Enteral and Parenteral Nutrition in Critically Ill Adult Patients in the Intensive Care Unit: An Overview of Systematic Reviews and Meta-Analysis

**DOI:** 10.3390/jcm14030991

**Published:** 2025-02-04

**Authors:** Paraskevi Papanikolaou, Xenophon Theodoridis, Androniki Papaemmanouil, Niki N. Papageorgiou, Alexandra Tsankof, Anna-Bettina Haidich, Christos Savopoulos, Konstantinos Tziomalos

**Affiliations:** 1 First Propaedeutic Department of Internal Medicine, University General Hospital of Thessaloniki AHEPA, Aristotle University of Thessaloniki, 54636 Thessaloniki, Greece; atsankof@auth.gr (A.T.); csavvopo@auth.gr (C.S.); ktziomal@auth.gr (K.T.); 2 Laboratory of Hygiene, Social and Preventive Medicine and Medical Statistics, School of Medicine, Faculty of Health Sciences, Aristotle University of Thessaloniki, 54124 Thessaloniki, Greece; xtheodoridis@auth.gr (X.T.); acpapaem@auth.gr (A.P.); npapaga@auth.gr (N.N.P.); haidich@auth.gr (A.-B.H.)

**Keywords:** enteral nutrition, parenteral nutrition, nutrition therapy, critically ill, ICU, overview of systematic review

## Abstract

**Background/Objectives**: Uncertainty persists about the best methods and timing for providing medical nutrition therapy (MNT) in the acute phase of critical illness. We conducted an overview of systematic reviews to examine and appraise the findings of the current systematic reviews and performed an updated meta-analysis incorporating newly published randomized controlled trials (RCTs) to investigate whether enteral nutrition (EN) is superior to the combination of EN and parenteral nutrition (PN) in patients admitted to the intensive care unit (ICU). **Methods**: We systematically searched three databases to retrieve systematic reviews and RCTs. Two independent reviewers performed the screening, data extraction, and quality assessment processes. The random effects model was utilized to synthesize the data regarding primary and secondary outcomes. **Results**: There was no difference between the two interventions regarding the efficacy and safety endpoints, apart from the bloodstream infections, which were found to be increased in the group that received the combination of EN+PN (RR = 1.27, 95%CI = 1.03 to 1.56, PI = 0.91 to 1.77, I^2^ = 0%). **Conclusions**: According to the present overview of systematic reviews and meta-analyses, there was no observed benefit on mortality, length of ICU stay or hospitalization, and duration of mechanical ventilation in critically ill patients receiving a combination of EN and PN in comparison to those receiving sole enteral nutrition in the ICU. Furthermore, no difference was observed in the rates of respiratory infections as well as the appearance of adverse events, such as vomiting and diarrhea. On the other hand, there was an increase in bloodstream infection rates in patients who received EN+PN compared to EN alone. Due to the limited implications of the results in clinical practice, further research is needed.

## 1. Introduction

Malnutrition in the intensive care unit (ICU) is associated with poor clinical outcomes and increased risk of mortality [1]. This could be attributed to the increased catabolic rate related to acute or chronic diseases, trauma, systemic inflammatory response syndrome (SIRS), multi-organ dysfunction syndrome (MODS), or the coexistence of malignancies [2,3,4]. The prevalence of malnutrition in the ICU fluctuates between 38% and 78% due to the increased rate of weight loss related to impaired nutritional status [5]. Critically ill patients staying more than 48 h in the ICU are considered at high malnutrition risk [1].

Medical nutrition therapy (MNT) plays a pivotal role in survival and prevents muscle mass degradation by providing an adequate amount of energy and protein. Particularly in the ICU, MNT seeks to prevent malnutrition in patients who are initially well-nourished and to prevent further worsening in those already experiencing malnutrition [6]. In light of this observation, MNT shall be considered for all ICU patients, especially those who will stay more than 48 h in the ICU [1]. To meet individual energy and protein needs in critically ill patients with mechanical ventilation support, the use of indirect calorimetry should be considered [1]. If indirect calorimetry or VO_2_ or VCO_2_ measurements are unavailable, simple weight-based equations (e.g., 20–25 kcal/kg/day) may be the preferred method [1,7].

According to international guidelines, MNT encompasses direct oral feeding and oral nutritional supplements (ONS) as the preferred option [8]. Alternatively, enteral nutrition (EN) should be used if the patients have a functioning gastrointestinal tract and are not able to ingest food orally [9,10,11,12]; otherwise, parenteral nutrition (PN) is needed, both methods should be delivered within 24–48 h of ICU admission [13,14,15]. For patients requiring enteral tube feeding for an estimated duration of less than four weeks, EN via a nasogastric or nasojejunal tube is recommended. However, if the anticipated need for nutritional support exceeds four weeks, a percutaneous endoscopic gastrostomy (PEG) or a percutaneous endoscopic gastrojejunostomy (PEG-J) is preferred [16]. Parenteral nutrition, whether in the form of total parenteral nutrition (TPN), as a complement to EN, or as supplemental parenteral nutrition (SPN), is used as an alternative when oral feeding is contraindicated, not well tolerated, or inadequate [17]. Systemic contraindications of EN rely on the clinical status of the patients which precludes feeding catheter placement. Mechanical contraindications are those when the placement of a feeding tube is not indicated in the specific condition, which may be altered in the future, such as hepatomegaly or previous abdominal surgery. Absolute contraindications include mechanical intestinal obstruction, active peritonitis, uncorrectable coagulopathy, bowel ischemia, and traumatic injuries of the head and neck [18,19].

EN alone may not adequately meet the energy and protein requirements of patients admitted to the ICU, especially during the first week of hospitalization. Furthermore, complications associated with EN are notable, with reported rates including 20% for vomiting, 1 to 4% for aspiration, and 2% to 63% for diarrhea [20]. Conversely, PN derived bloodstream fungal infections appeared in 4%, compared to those who did not receive PN [21]. Hepatotoxicity, cholecystitis (observed in 25% of cases), hyperglycemia, and overfeeding have also been observed [22,23]. The European Society for Clinical Nutrition and Metabolism (ESPEN) recommends SPN initiation on a case-by-case basis when a full dose of EN is not tolerated in the first week of ICU stay, while full dosages of EN and PN may be avoided within the first 3 days to prevent overfeeding [1]. On the other hand, the American Society for Parenteral and Enteral Nutrition (ASPEN) does not recommend SPN administration earlier than 7 days of ICU admission to non-malnourished patients, as the average critically ill patient is unlikely to experience adverse effects from delaying SPN administration for one week. Additionally, the patient’s ability to tolerate EN may improve during this period. However, the initiation of SPN within the first week of hospitalization in malnourished patients and those with diminished lean muscle mass relies on clinicians’ judgment [24].

Recent systematic reviews (SRs) and meta-analyses comparing the co-administration of EN and PN in ICUs show no difference in mortality rates and hospitalization or duration of mechanical ventilatory support but indicate an improvement in nutritional and immunological status [25,26,27]. On the other hand, three studies support the claim that the co-administration of EN and PN reduces mortality risk and nosocomial infections [27,28,29].

Published systematic reviews and meta-analyses reported inconclusive results regarding this clinical question due to their lack of methodological rigor, the small number of available clinical trials, and the heterogeneity among participants’ diagnoses, timing of nutritional administration, and dosing of MNT. Additionally, the existing analyses do not distinguish between the two different administration strategies, EN early combined with PN or SPN to EN after a certain duration when the latter fails to meet nutritional requirements. To shed light on this clinical question, we conducted an overview of systematic reviews to examine and appraise the findings of the current systematic reviews and performed an updated meta-analysis incorporating newly published randomized controlled trials (RCTs) to investigate whether EN is superior to EN+PN in patients admitted to the ICU.

## 2. Materials and Methods

The present overview of systematic reviews follows the Preferred Reporting Items for Overviews of Reviews (PRIOR) guidelines (Supplementary Table S1) [30], while the meta-analysis is by the Items for Systematic Reviews and Meta-Analyses (PRISMA) guidelines (Supplementary Table S2) [31]. The study protocol registration ID is CRD42024588342 at PROSPERO.

### 2.1. Search Strategy and PICO

A systematic literature search was conducted in PubMed, Cochrane Central, and SCOPUS from 2000 to 2024 for eligible systematic reviews and meta-analyses comparing the administration of EN in ICU critically ill adult patients in comparison with EN+PN (Supplementary Table S3). Furthermore, a second search on the same database was conducted for eligible RCTs (Supplementary Table S3). Search terms such as “enteral nutrition”, “enteral feed”, “parenteral feed”, “parenteral nutrition”, “artificial feed*”, “supplementary parenteral nutrition”, “intravenous supplementation”, “ICU”, “critical care”, etc. were used and adjusted to each database. Reference lists of the included studies and gray literature were reviewed for relevant studies. Finally, an expert in systematic review methodology was consulted to identify potentially eligible studies. Additionally, ClinicalTrials.gov was searched for ongoing studies in this field. The search strategy was based on the PICO acronym (participants: adult critically ill patients in ICU; intervention: EN and PN; control: EN; outcomes: overall mortality, length of ICU stay/mechanical ventilation, etc.). Full texts that could not be obtained after contacting the study authors were excluded.

### 2.2. Study Selection, Inclusion, and Exclusion Criteria

The study selection process for systematic reviews and/or meta-analyses was conducted by two independent reviewers (P.P. and A.P.), and a third one (X.T.) was consulted to solve any conflict. The selection process was conducted with the use of the Rayyan screening tool [32]. Duplicates were removed, the title and abstract of the remaining studies were examined, and the irrelevant studies, according to our inclusion criteria, were excluded. The full text of the remaining studies was evaluated, and the most relevant to the topic of systematic reviews and/or meta-analyses were included in the overview.

Similarly, two independent reviewers (P.P. and A.P.) selected the most relevant RCTs for the meta-analysis. With the use of the Rayyan screening tool, the duplicates were removed, the titles and the abstracts of all clinical trials were screened, and those that were irrelevant to the topic were excluded. The full text of those remaining was examined, and the most relevant RCTs were included in the meta-analysis. A third reviewer (X.T.) was conducted to resolve any disagreement.

Studies were included in the overview of systematic reviews if they met the following criteria: (1) Systematic reviews and/or meta-analyses including RCTs, published in English between 2000 and 2024; (2) Participants were adult, critically ill patients hospitalized in an ICU with acute or chronic diseases, trauma, burns, malignancy, and multi-organ failure and after a major surgery; (3) Intervention: Combination of enteral and parenteral nutrition, or any intravenous co-administration of nutrient supplements; or (4) Comparison: enteral nutrition (Co-administration of nutrient supplements would be acceptable in the case this intervention would also be applied in the intervention group.).

Eligible RCTs for the meta-analysis fulfilled the following criteria: 1. Randomized control trials, published in English between 2000 and 2024; 2. Participants were adult, critically ill patients hospitalized in an ICU with acute or chronic diseases, trauma, burns, malignancy, and multi-organ failure and after major surgery; 3. Intervention: Combination of enteral and parenteral nutrition, or any intravenous co-administration of nutrient supplements; or 4. Comparison: enteral nutrition (Co-administration of nutrient supplements would be acceptable in the case this intervention would also be applied in the intervention group.).

The outcomes of interest were categorized as primary and secondary. Primary outcomes were all-cause mortality, mortality at 30 days, length of stay in ICU and hospital, and duration of mechanical ventilation. In-hospital infections (respiratory infection and bloodstream infection), inflammatory markers (albumin, procalcitonin, C-reactive protein), and adverse effects (vomiting, hyperglycemia, diarrhea, hepatotoxicity) were considered as secondary outcomes.

In contrast, this study excluded observational studies, studies on pediatric populations or animals, and studies not published in English.

### 2.3. Data Extraction

Data extraction was conducted and summarized in a piloted standardized data collection form by two reviewers (P.P. and A.P.) who worked independently, while a third one (X.T.) was consulted to resolve any conflict. Extracted information from the systematic reviews consisted mainly of studies’ characteristics, year of publication, PICO, number of included RCTs and participants, and results of main outcomes (Table 1). Data that were collected from the RCTs (Table 2) were authors’ names, year of publication, country, the design of the study, participants’ characteristics, number of patients in each group, intervention and comparison type, study duration, measured variables, and side effects.

### 2.4. Corrected Cover Area

The corrected cover area (CCA) formula was used to calculate the percentage of overlap among the primary studies between the included systematic reviews [47,48]. Values between 0% and 5% correspond to slight overlap; values between 6% and 10% indicate moderate overlap; values between 11% and 15% indicate high overlap; and values greater than 15% correspond to a very high overlap [49,50].

### 2.5. Risk of Bias, Quality Assessment, and Quality of Evidence

Two independent reviewers (P.P. and N.N.P.) evaluated the included studies. Any disagreement was resolved by a third reviewer (X.T.). The quality of included systematic reviews was conducted according to the AMSTAR 2 tool [51] and the quality of the included RCTs based on the revised risk of bias tool (RoB 2.0) [52], according to Cochrane Handbook principles. The Robvis tool was used for figure formation [53].

The AMSTAR 2 tool is composed of 16 domains evaluating the PICO, adjustment to protocol, the methodological conduction of the review, the statistical methods that were used, and possible heterogeneity and publication bias impact on the confidence of the results.

Based on the RoB 2.0 tool assessment, a study was characterized to be at low risk of bias when all domains were considered of low risk of bias, at some concerns when at least one out of the five domains graded with some concerns but not to be at high risk for any domain, and at high risk of bias when at least three out of five domains evaluated at some concerns or at least one domain received a high risk of bias [52].

Finally, the grading of recommendations assessment, development, and evaluation (GRADE) was used to evaluate the certainty of present meta-analysis’ findings, characterizing them as of high, moderate, low, or very low certainty [54].

### 2.6. Statistical Analysis

Continuous outcomes were summarized using mean differences (MDs) and their 95% confidence intervals (CI), while dichotomous data were analyzed and presented as risk ratio and 95%CIs. When the median, interquartile range, and range were reported, the mean and standard deviation were calculated by the use of the Wan formula [55,56]. Due to potential heterogeneity between studies, the random effects model was used to pool the effect size of our studies. Heterogeneity was assessed with the I² statistic, referring to the percentage of volatility in the effect of the actual values due to heterogeneity and not due to random error. The scaling of I^2^ is as follows: 0–40% indicating clinically insignificant heterogeneity, 30–60% for moderate heterogeneity, 50–90% in case of significant heterogeneity, and 75–100% characterizing very high heterogeneity [57].

If feasible (≥10 studies), publication bias was assessed by generating funnel plots and using Egger’s test [58]. In case of missing data, authors of the primary studies were contacted. All analyses were performed in R Studio software.

## 3. Results

### 3.1. Study Selection Process and Main Characteristics of Included Studies

A total of 10,585 records were retrieved. After removing duplicates and irrelevant content, a total of 146 articles were reviewed, and five systematic reviews and meta-analyses [25,26,27,28,29] (Table 1) were eligible according to the inclusion criteria (Supplementary Figure S1). Following the same criteria, 2480 RCTs were identified after exclusions, and a total of 94 studies were examined in full text. Finally, a total of 14 RCTs [33,34,35,36,37,38,39,40,41,42,43,44,45,46] (Table 2) were included in the updated meta-analysis. Supplementary Tables S4 and S5 present the detailed reasons for the exclusion of the systematic reviews and RCTs, respectively.

### 3.2. Overlap of OoSR and Corrected Covered Area

We retrieved five systematic reviews including a total of 14 primary studies. The review by Hill et al. [25] included two unique primary studies, while in total covered 12 studies. Both reviews of Alsharif et al. [29] and Dhaliwal et al. [26] included 5 studies, while reviews conducted by Shi et al. [27] and Lewis et al. [28] included 8 studies each, with no unique primary studies. The overview of systematic reviews showed a high overlap rate of 0.19 (19.35%), while the corrected covered area, adjusted for structural zeros, was 24.49% (Supplementary Figure S2).

### 3.3. Risk of Bias Assessment of the Included Systematic Reviews

According to the AMSTAR 2 tool, two SRs were classified as “low overall quality”, two appeared as “very low overall quality”, while one study achieved “high overall quality” (Supplementary Table S6). The downgrading was due to a lack of information on possible publication bias, funding sources of the included RCTs, and inadequate selection of primary studies.

### 3.4. Risk of Bias Assessment of the Included RCTs

**Figure 1:**
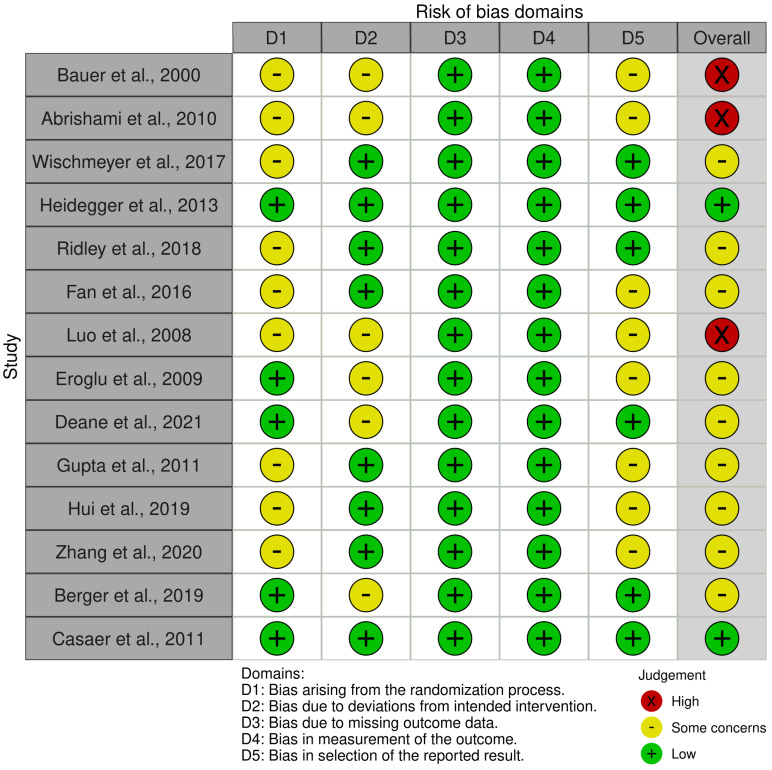
Risk of bias assessment of the included randomized controlled trials [33,34,35,36,37,38,39,40,41,42,43,44,45,46].

Of the 14 studies, two trials were classified as having a “low risk of bias” [36,40], nine studies were considered to have “some concerns” [35,37-39,41,43-46], and the remaining three RCTs appeared to have a “high risk of bias” [33,34,42] due to the absence of a pre-specified protocol and information regarding the allocation concealment of the included RCTs (Figure 1).

### 3.5. Effects of Enteral and Parenteral Nutrition Co-Administration Compared to Sole Enteral on Mortality, Days in ICU/Hospital, and Duration of Ventilation

Twelve studies evaluated the overall mortality of the patients (Supplementary Figure S3), and only two evaluated the mortality on day 30 (Supplementary Figure S4), with no difference being observed between the two study arms (RR = 0.89, 95%CI = 0.72 to 1.11, PI = 0.59 to 1.35, I^2^ = 16% and RR = 0.66, 95%CI = 0.42 to 1.01, I^2^ = 0%, respectively). Furthermore, the days of overall hospitalization (MD = 0.48, 95%CI = −2.40 to 3.36, PI = −7.88 to 8.83, I^2^ = 88%) (Supplementary Figure S5) and LOS in the ICU (MD = −0.19, 95%CI = −1.19 to 0.80, PI = −2.68 to 2.29, I^2^ = 53%) (Supplementary Figure S6) were not influenced by the type of intervention. The significant variability in hospitalization duration may be attributed to the diverse clinical diagnoses of patients and their corresponding medical treatments. Additionally, the duration of mechanical ventilation support (Supplementary Figure S7) was estimated in ten primary studies, presenting no difference between groups (MD = −0.52, 95%CI = −1.38 to 0.33, PI = −2.56 to 1.51, I^2^ = 35%).

### 3.6. Effect of the Intervention on Secondary Outcomes

Seven of the primary studies evaluated respiratory infections (Supplementary Figure S8), and five studies assessed bloodstream infections (Supplementary Figure S9), with no observed differences for respiratory infections but with an increased risk of bloodstream infection for the combination of EN+PN (RR = 1.05, 95%CI = 0.84 to 1.31, PI = 0.63 to 1.74, I^2^ = 28%, and RR = 1.27, 95%CI = 1.03 to 1.56, PI = 0.91 to 1.77, I^2^ = 0%, respectively). Adverse events, i.e., vomiting (Supplementary Figure S10) and diarrhea (Supplementary Figure S11), were assessed by two and four studies, respectively, showing no difference regarding vomiting and diarrhea rates (RR = 1.40, 95%CI = 0.65 to 3.01, I^2^ = 0%, and RR = 0.80, 95%CI = 0.36 to 1.76, PI = 0.02 to 25.87, I^2^ = 87%, respectively).

According to the findings from the serological markers, no effect was observed between the two interventions. More specifically, albumin (MD = −0.44 g/L, 95%CI = −3.68 to 2.80, PI = −15.50 to 14.62, I^2^ = 91%) and glucose (MD = −1.56 mg/dL, 95%CI = −14.38 to 11.25, PI = −84.64 to 81.52, I^2^ = 0%) levels were not influenced by the EN+PN administration. Additionally, CRP concentrations (MD = −3.46 mg/L, 95%CI = −33.22 to 26.30, PI = −349.68 to 342.76, I^2^ = 75%) were not affected by the type of nutritional intervention (Supplementary Figures S12-S14). No changes in serological markers were observed due to the limited data from a few small-sample RCTs. A further pre-specified analysis, as outlined in the protocol, regarding procalcitonin levels—a marker indicative of inflammation within 24 h—and hepatotoxicity was not conducted due to insufficient data in the primary studies.

### 3.7. Certainty of Evidence

According to the GRADE methodology, the certainty of evidence for both primary and secondary outcomes was assessed as low or very low (Supplementary Table S7). Notably, most outcomes were downgraded due to concerns related to the domains of risk of bias and indirectness.

### 3.8. Publication Bias

We evaluated the presence of publication bias in comparisons where ≥10 studies were included. Publication bias was not detected for total mortality (*p* = 0.3383) and duration of mechanical ventilation (*p* = 0.3904). However, publication bias was indicated for ICU LOS (*p* = 0.0044) due to the smaller number of participants in the included RCTs. Thus, additional clinical trials with larger patient samples are needed to confirm this finding.

## 4. Discussion

In the present overview, we included five systematic reviews and meta-analyses, with a total of 17,232 participants. Three reviews [25,26,27] showed no effect on overall mortality, length of hospitalization or ICU stay, or mechanical ventilation support days, while two [28,29] showed a decreased mortality incidence in the intervention group. On the other hand, one study [29] presented a decreased incidence of nosocomial infections in the intervention group, while Shi et al. [27] supported that the EN group had fewer respiratory infections.

In the present meta-analysis, we included 14 RCTs, with 5808 critically ill patients in the ICU, assessing the efficacy of the co-administration of EN+PN compared to sole EN on overall mortality, mortality at 30 days, length of ICU and hospital stay, duration of mechanical ventilation support, as well as the establishment of adverse events, such as respiratory or bloodstream infections, diarrhea and vomiting, and changes of inflammatory and serological markers. No effect was observed on mortality rates, length of ICU stay, and overall hospitalization, as well as the duration of mechanical ventilation support. Only a few studies referred to the effect of combined EN and PN in adverse events such as respiratory and bloodstream infections, inflammatory markers’ changes, glycemic control, or gastrointestinal adverse events, such as vomiting and diarrhea’s presentation, and the analysis indicated no difference apart from the bloodstream infections, which were increased in the EN+PN group. Analysis of procalcitonin levels and the development of hepatotoxicity were unable to be conducted due to a lack of existing data.

We found no difference in overall mortality, including mortality at 30 days, between the two groups. Our findings regarding the mortality at 30 days are in agreement with the meta-analyses conducted by Hill and colleagues [25]. The systematic review by Dhaliwal et al. [26] also showed that the two groups did not differ on this outcome; however, they define mortality as ICU and hospital mortality. Finally, Shi and colleagues evaluated [27] the hospital mortality, and their meta-analysis indicated that the combination of EN+PN did not differ compared to the provision of EN alone. On the other hand, Alsharif and colleagues [29] found that the combination of SPN+EN was superior to EN alone regarding ICU mortality. This discrepancy may be due to their focus on ICU mortality and their inclusion of only four studies. Additionally, Lewis and colleagues [28] showed that the combination of EN+PN had a beneficial effect on mortality at 30 days. This disagreement may be attributed to the fact that it included one study, in which it is not clearly stated whether mortality refers to 30 days, as well as an older study with a small sample size, which was excluded from our review according to the eligibility criteria.

As far as days of hospitalization and ICU LOS are concerned, our findings indicated that there was no difference between the two groups. The findings from all but one included meta-analysis [25,26,27,29] that assessed these outcomes are in line with our observation.

The combination of EN+PN was not superior to EN alone in improving the duration of mechanical ventilation of ICU patients. The included systematic reviews and meta-analyses by Alsharif and colleagues [29], Hill and colleagues [25], and Shi and colleagues [27] reached the same conclusion.

In the present systematic review and meta-analysis, we also evaluated the effect of the two groups on adverse events. We found that EN+PN was associated with increased risk of bloodstream infections of the patients admitted to the ICU. On the contrary, we did not find a difference between the two groups regarding the remaining studied adverse events, such as nosocomial respiratory infections, diarrhea, and vomiting. The meta-analysis conducted by Alsharif and colleagues [29] found that the combination of EN+PN had a beneficial effect on the presence of infections. However, it should be noted that they did not clearly define infections, which may lead to the inclusion and analysis of different types of infections, such as respiratory, urinary tract, wound, etc. The systematic review by Lewis and colleagues [28] found that there was no difference between the two groups regarding bloodstream infections. This discrepancy may be attributed to the fact that their meta-analysis included only two randomized controlled trials (RCTs), whereas our analysis incorporated five RCTs. Consequently, our overall analysis benefits from a larger sample size.

Regarding albumin concentrations, we showed that the combination of EN+PN did not have an effect in comparison with EN alone. Our results are in line with the findings by Shi and colleagues [27], which was the only systematic review that also investigated this outcome of interest. Contrary to that, none of the included systematic reviews evaluated the effect of EN+PN compared to sole EN on CRP concentrations.

Our results showed no difference in respiratory occurrence and are in contrast with Alsharif et al.’s study [29] and Dhaliwal et al.’s study [26], which both claim reduction of nosocomial infections due to EN and PN co-administration, although it should be mentioned that in the meta-analyses only five RCTs were included, with different types of nosocomial infections. On the other hand, Shi et al. [27] support that EN is beneficial by reducing respiratory infections establishment.

The implications of our results for research strengthen the pre-existing statement that the combination of EN and PN is not superior to sole EN administration in critically ill patients in the ICU, as no statistically significant alterations are observed in mortality incidence, hospitalization, and duration of mechanical ventilation support. Thus, the co-administration of EN and PN cannot be applied to usual practice, but only when sole EN is not tolerated, is insufficient, or is contraindicated, as it may improve the nutritional status but not the overall clinical outcome. Furthermore, the financial cost of PN is significantly higher than that of EN solutions.

Although this systematic review and meta-analysis followed PRISMA and PRIOR instructions, some limitations should be acknowledged. The eligible studies were few, including a small number of participants and yielding high clinical heterogeneity due to the variety of medical diagnoses at the time of ICU admission. Thus, the present clinical question should be answered in the future by conducting RCTs with a greater number of participants, categorized according to their main diagnosis in the ICU, as well as their coexisting diseases and current medical treatment that may interfere with the nutrition treatment provided. Further, future studies comparing disease-specific types of EN and PN regimens, based on the patient’s diagnosis in comparison with standard-type solutions, will be beneficial. Also, further research on the impact of EN or PN and their combination on serological markers, such as procalcitonin and liver enzymes, to assess hepatotoxicity will be valuable for the patients’ optimal medical management.

## 5. Conclusions

According to the present overview of systematic reviews and meta-analyses, there was no observed benefit on mortality, length of ICU stay or hospitalization, and duration of mechanical ventilation in critically ill patients receiving a combination of EN and PN in comparison to those receiving sole enteral nutrition in the ICU. Furthermore, no difference was observed in the rates of respiratory infections as well as the appearance of adverse events, such as vomiting and diarrhea. However, there was an increase in bloodstream infection rates in patients who received EN+PN compared to EN alone. More randomized controlled trials with robust methodology are needed to increase the certainty of the evidence on this topic.

## Figures and Tables

**Table 1:** Characteristics of the included systematic reviews and meta-analyses.

	Alsharif et al. [29]	Dhaliwal et al. [26]	Hill et al. [25]	Lewis et al. [28]	Shi et al. [27]
Publication Year	2020	2004	2021	2019	2018
Journal	*Nutrients*	*Intensive Care Med*	*JPEN J Parenter Enteral Nutri*	*The Cochrane Database Syst Rev*	*Medicine*
Protocol Registration	CRD42019121888	NR	CRD42020184355	CD012276 *	NR
PICO	P: Critically ill ≥16 y ICU I: SPN+EN C: EN O: Mortality, LOS in ICU/ hospitalization, stay at mechanical ventilation, nosocomial infections	P: Critically ill adults in ICU I: EN+PN C: EN O: Mortality, nosocomial infections, nutritional intake	P: Critically ill >18 y in ICU I: EN+PN C: EN O: Mortality, hospitalization, stay at mechanical ventilation	P: Critical ill adults >16 yo in ICU > 24 h, with trauma/medical/ postsurgical I: EN and EN+PN C: EN O: Mortality in hospital (30 d, 90 d, 180 d), ICU/ventilator-free days, GI adverse events	P: Critically ill adults in ICU I: EN+PN C: EN O: Mortality, LOS in ICU/hospitalization, stay at mechanical ventilation, respiratory infections
Searched Databases	PubMed, Embase, CENTRAL	Medline, Embase, Cochrane Library	Medline, Embase, CENTRAL	CENTRAL, MEDLINE, Embase	PubMed, EMBSE, Cochrane library
Search Period	January 1990 to January 2019	1980 to 2003	Inception to 8 May 2020	1946 to 3 October 2017	Until June 2018
Language Restriction	Only English	Not referred	No restriction	No restriction	No restriction
Number of Included Studies	Qualitative analysis-7 RCTs Quantitative analysis-5 RCTs (scored > 3 Jadad score)	5 RCTs	12 RCTs	8 RCTs	8 RCTs
Number of Participants	698	233	5543	5398	5360
Quality Assessment of Included Studies	Jadad	Experts’ tool based	RoB tool	RoB tool	RoB tool
Publication Bias Assessment	NR	NR	NR	Funnel plot	NR
Sensitivity Analysis	NR	NR	In two trials because SPN provided to both intervention and control group.	Conducted on the outcomes of mortality, ICU free days and ventilation days.	Conducted after the removal of one trial due to high heterogeneity in the result of LOS ICU.
Funding	Deputyship for Research and Innovation, Ministry of Education Saudi Arabia	NR	Baxter Health Care Corporation	NIHR Cochrane Collaboration Programme Grant	Natural science foundation of Guangxi province
Main Finding	EN+SPN 1. Increases energy and protein intake. 2. Decreases nosocomial infections/ICU mortality.	EN+PN 1. No effect in mortality/ nosocomial infections. 2. Increases nutritional intake.	EN+PN 1. No reduction in mortality/LOS and duration of mechanical ventilation. 2. Increases nutrition intake.	EN+PN 1. Reduce mortality/ventilator days and adverse effects.	EN+PN 1. No effect on outcomes. 2. EN decreases respiratory infections and LOS in hospital.
Certainty of Evidence	NR	NR	NR	GRADE	NR
AMSTAR 2	Critical Low	Low	Low	High	Critical Low

CENTRAL: Cochrane Central Register of Controlled Trials; CINAHL: Cumulative Index to Nursing and Allied Health Literature; CRD: Centre for Reviews and Dissemination; EN: enteral nutrition; GI: gastrointestinal; GRADE: grading of recommendations assessment, development, and evaluation; ESPEN: European Society for Clinical Nutrition and Metabolism; ICU: intensive care unit; LOS: length of stay; NR: not reported; PN: parenteral nutrition; RCT: randomized controlled trials; SR: systematic reviews. * Published in the Cochrane Database in 2016. Later, the lead author based on this protocol made changes (title, intervention, and control) for the final review.

**Table 2:** Characteristics of the included randomized controlled trials.

Study ID	Protocol Registration	Population	Intervention	Comparator	Main Findings
Abrishami et al., 2010 [33]	NR	Patients ≥ 18 years old, ICU admission (<24 h), having SIRS, APACHE II score > 10 and expected not to feed via oral route for at least 5 days.	PN+EN	EN	No difference in regard to inflammation, while severity of illness may not change with these regimens.
Bauer et al., 2000 [34]	NR	Patients > 18 years old, admitted to ICU > 2 days, expected to eat < 20 kcal/kg/d > 2 days, and EN to be progressively administered for >2 days.	PN+EN	EN	EN+PN enchases nutrient intake and corrects nutritional parameters (prealbumin) within 1 week, supplemental PN has no clinically relevant effect at the early phase of nutritional support.
Berger et al., 2018 [35]	NCT02022813	Mechanically ventilated patients received till day 3 < 60% of the equation target (25 kcal/kg * day) by EN alone, and expected to require >5 days of ICU therapy.	SPN+EN	EN	SPN+EN from D4 was associated with improved immunity, less systemic inflammation and a trend to less muscle mass loss.
Casaer et al., 2011 [36]	NCT00512122	Adult patients, NRS ≥3 *	EN+EPN	LPN	LPN was associated with faster recovery and fewer complications, as compared with EPN.
Eroglu, 2009 [37]	NR	Adults, aged 18 to 65 yr with a severe multiple injury based on an ISS > 20.	EN+IV alanyl-glutamine	Control **	Intervention for 7 days increases total plasma glutathione.
Fan et al., 2016 [38]	NR	Patient in NICU with the diagnosis of STBI if: (1) GCS score: 6–8; (2) NRS ≥ 3 *.	EN+PN	EN	EN+PN promote the recovery of the immune function, enhance nutritional status, decrease complications and improve the clinical outcomes in patients with severe traumatic brain injury.
Gupta et al., 2011 [39]	NR	Patients with bilateral pulmonary infiltrates in the chest radiograph, PaO_2_/FiO_2_ < 200, and pulmonary capillary pressure <18 mm Hg.	EN+PN Omega 3 fatty acids	EN	In ventilated patients with ARDS, intravenous Omega 3 fatty acids alone do not improve ventilation, length of ICU stay, or survival.
Heidegger et al., 2013 [40]	NCT00802503	Patients received <60% of their energy target from EN at day 3 after admission to the ICU, were expected to stay for >5 days, expected to survive for >7 days, and had a functional gastrointestinal tract.	EN+SPN	EN	SPN starting 4 days after ICU admission could reduce nosocomial infections.
Hui et al., 2019 [41]	NR	Patients diagnosed with severe acute pancreatitis (SAP).	EN+TPN	EN	Early enteral nutrition could improve nutritional status, shorten the course of the disease, and reduce the incidences of infection, death, and complication, but increase the risk of abdominal distension and regurgitation.
Luo et al., 2008 [42]	NR	Subjects of 18 to 90 years of age with functional access for enteral tube feeding and requiring non-elemental tube feeding for at least 8 days.	EN+IV Ala-glutamine	EN	Alanyl-Gln administration by enteral or parenteral routes did not affect antioxidant capacity or oxidative stress markers, gut barrier function, or whole-body protein metabolism compared to control.
Ridley et al., 2018 [43]	NCT01847534	Patients ≥ 16 years old, stayed in ICU 48–72 h, under MV, with organ failure.	EN+SPN	EN	EN+SPN, applied over 7 days, significantly increased energy delivery when compared to EN. Clinical and functional outcomes were similar between the two patient groups.
Wischmeyer et al., 2017 [44]	NCT01206166	Patients >18 years old in the ICU were considered eligible if: (1) require MV > 72 h, (2) receiving EN or were to be initiated on EN within 48 h of ICU admission (3) had a BMI of <25 or >35.	EN+SPN	EN	No significant outcome differences were observed between groups, including no difference in infection risk. Potential, although statistically insignificant, trends of reduced hospital mortality in the SPN+EN group versus the EN-alone group were observed.
Deane et al., 2021 [45]	ACTRN 1261900 0121167	Patients of >18 years old with a reduction serum phosphate to 0.65 mmol/L	EN+IV thiamine	EN	No significant decrease in mortality, blood lactate levels and days of vasopressor medication administration were observed in both groups.
Zhang et al., 2020 [46]	NR	Patients with craniocerebral injury, with GCS within 8 points, and requiring to be hospitalized for nutrition support for >2 weeks.	EN+PN	EN	EN+PN can improve the nutritional status of patients, contribute to the recovery of cellular immune function, reduce complications and promote the repair of neurological function.

APACHE II: acute physiology and chronic health evaluation; ARDS: acute respiratory distress syndrome; BMI: body mass index; D4: day 4; EN: enteral nutrition; EPN: early parenteral nutrition; ICU: intensive care unit; ISS: injury severity score; IV: intravenous; GCS: Glasgow coma scale; LPN: late parenteral nutrition; MV: mechanical ventilation; NICU: neurosurgery intensive care unit; NR: not referred; NRS 2002: nutrition risk score; SIRS: systemic inflammatory response syndrome; SPN: supplemental parenteral nutrition; STBI: severe traumatic brain injury. * NRS ≥ 3: at risk of malnutrition; Control ** solution without alanyl-glutamine.

## Data Availability

The original contributions presented in this study are included in the article/Supplementary Material. Further inquiries can be directed to the corresponding author(s).

## Supplementary Materials

The following supporting information can be downloaded at https://www.mdpi.com/article/10.3390/jcm14030991/s1: Table S1: PRIOR checklist; Table S2: PRISMA checklist; Table S3: Search strategy; Table S4: Excluded systematic reviews; Table S5: Excluded randomized controlled trials; Table S6: AMSTAR 2.0 tool; Table S7: GRADE; Figure S1: PRISMA flow diagram; Figure S2: Corrected covered area; Figures S3–S7: Effect of EN+PN on primary outcomes; Figures S8–S14: Effect of EN+PN on secondary outcomes.
