# Supplementary material for: Enteral Nutrition Versus a Combination of Enteral and Parenteral Nutrition in Critically Ill Adult Patients in the Intensive Care Unit: An Overview of Systematic Reviews and Meta-Analysis
Source: J Clin Med. 2025 Feb 4;14(3):991. doi: 10.3390/jcm14030991 (PMC11818211; doi:10.3390/jcm14030991)

# Contents

**Supplementary Table S1:** PRIOR checklist for the Preferred Reporting Items for Overviews of Reviews

**Supplementary Table S2:** PRISMA checklist for Preferred Reporting Items for Systematic Reviews and Meta-Analyses

**Supplementary Table S3**: Search strategy for identifying systematic reviews in the PubMed database

**Supplementary Table S4**: Detailed reasons for exclusion of the systematic reviews

**Supplementary Table S5**: Detailed reasons for exclusion of the randomized controlled trials

**Supplementary Table S6**: AMSTAR 2.0 tool for the assessment of the included systematic reviews

**Supplementary Table S7**: Assessment of certainty of evidence according to GRADE

**Supplementary Figure S1**: PRISMA flow diagram for the selection process of the systematic reviews

**Supplementary Figure S2**: Corrected covered area and corrected covered area after adjusting by structural zeros, respectively

**Supplementary Figure S3**: Effect of EN+PN compared to EN alone on overall mortality

**Supplementary Figure S4**: Effect of EN+PN compared to EN alone on mortality in the ICU

**Supplementary Figure S5**: Effect of EN+PN compared to EN alone on hospitalization

**Supplementary Figure S6**: Effect of EN+PN compared to EN alone on ICU length of stay

**Supplementary Figure S7**: Effect of EN+PN compared to EN alone on mechanical ventilation support days

**Supplementary Figure S8**: Effect of EN+PN compared to EN alone on respiratory infections

**Supplementary Figure S9**: Effect of EN+PN compared to EN alone on bloodstream infections

**Supplementary Figure S10**: Effect of EN+PN compared to EN alone on vomiting

**Supplementary Figure S11**: Effect of EN+PN compared to EN alone on diarrhea

**Supplementary Figure S12**: Effect of EN+PN compared to EN alone on albumin (g/l)

**Supplementary Figure S13**: Effect of EN+PN compared to EN alone on glucose (mg/dl)

**Supplementary Figure S14**: Effect of EN+PN compared to EN alone on CRP (mg/l)

**Supplementary Table S1:** PRIOR checklist for the Preferred Reporting Items for Overviews of Reviews

| **Section** Topic | # | **Item** | **Location reported** |
|---|---|---|---|
| **TITLE** | | | |
| Title | 1 | Identify the report as an overview of reviews. | 1 |
| **ABSTRACT** | | | |
| Abstract | 2 | Provide a comprehensive and accurate summary of the purpose, methods, and results of the overview of reviews. | 1 |
| **INTRODUCTION** | | | |
| Rationale | 3 | Describe the rationale for conducting the overview of reviews in the context of existing knowledge. | 2 |
| Objectives | 4 | Provide an explicit statement of the objective(s) or question(s) addressed by the overview of reviews. | 2 |
| **METHODS** | | | |
| Eligibility criteria | 5a | Specify the inclusion and exclusion criteria for the overview of reviews. If supplemental primary studies were included, this should be stated, with a rationale. | 3-4 |
| | 5b | Specify the definition of 'systematic review' as used in the inclusion criteria for the overview of reviews. | NA |
| Information sources | 6 | Specify all databases, registers, websites, organizations, reference lists, and other sources searched or consulted to identify systematic reviews and supplemental primary studies (if included). Specify the date when each source was last searched or consulted. | 3 |
| Search strategy | 7 | Present the full search strategies for all databases, registers and websites, such that they could be reproduced. Describe any search filters and limits applied. | 3, Supplementary Table S3 |
| Selection process | 8a | Describe the methods used to decide whether a systematic review or supplemental primary study (if included) met the inclusion criteria of the overview of reviews. | 3-4 |
| | 8b | Describe how overlap in the populations, interventions, comparators, and/or outcomes of systematic reviews was identified and managed during study selection. | 3-4 |
| Data collection process | 9a | Describe the methods used to collect data from reports. | 4 |
| | 9b | If applicable, describe the methods used to identify and manage primary study overlap at the level of the comparison and outcome during data collection. For each outcome, specify the method used to illustrate and/or quantify the degree of primary study overlap across systematic reviews. | 4 |
| | 9c | If applicable, specify the methods used to manage discrepant data across systematic reviews during data collection. | NA |
| Data items | 10 | List and define all variables and outcomes for which data were sought. Describe any assumptions made and/or measures taken to identify and clarify missing or unclear information. | 4 |
| Risk of bias assessment | 11a | Describe the methods used to *assess* risk of bias or methodological quality of the included systematic reviews. | 4 |
| | 11b | Describe the methods used to *collect* data on (from the systematic reviews) and/or *assess* the risk of bias of the primary studies included in the systematic reviews. Provide a justification for instances where flawed, incomplete, or missing assessments are identified but not re-assessed. | 4 |
| | 11c | Describe the methods used to *assess* the risk of bias of supplemental primary studies (if included). | Figure 1 |
| Synthesis methods | 12a | Describe the methods used to summarize or synthesize results and provide a rationale for the choice(s). | 5 |
| | 12b | Describe any methods used to explore possible causes of heterogeneity among results. | 5 |
| | 12c | Describe any sensitivity analyses conducted to assess the robustness of the synthesized results. | 5 |
| Reporting bias assessment | 13 | Describe the methods used to *collect* data on (from the systematic reviews) and/or *assess* the risk of bias due to missing results in a summary or synthesis (arising from reporting biases at the levels of the systematic reviews, primary studies, and supplemental primary studies, if included). | NA |
| Certainty assessment | 14 | Describe the methods used to *collect* data on (from the systematic reviews) and/or *assess* certainty (or confidence) in the body of evidence for an outcome. | 5 |
| **RESULTS** | | | |

| **Section** Topic | # | Item | Location reported |
|---|---|---|---|
| Systematic review and supplemental primary study selection | 15a | Describe the results of the search and selection process, including the number of records screened, assessed for eligibility, and included in the overview of reviews, ideally with a flow diagram. | 5, Supplementary Figure S1 |
| | 15b | Provide a list of studies that might appear to meet the inclusion criteria, but were excluded, with the main reason for exclusion. | Supplementary Table S |
| Characteristics of systematic reviews and supplemental primary studies | 16 | Cite each included systematic review and supplemental primary study (if included) and present its characteristics. | Table 1 |
| Primary study overlap | 17 | Describe the extent of primary study overlap across the included systematic reviews. | 6, Supplementary Figure S2 |
| Risk of bias in systematic reviews, primary studies, and supplemental primary studies | 18a | Present assessments of risk of bias or methodological quality for each included systematic review. | 6, Supplementary Table S4 |
| | 18b | Present assessments (collected from systematic reviews or assessed anew) of the risk of bias of the primary studies included in the systematic reviews. | Figure 1 |
| | 18c | Present assessments of the risk of bias of supplemental primary studies (if included). | Figure 1 |
| Summary or synthesis of results | 19a | For all outcomes, summarize the evidence from the systematic reviews and supplemental primary studies (if included). If meta-analyses were done, present for each the summary estimate and its precision and measures of statistical heterogeneity. If comparing groups, describe the direction of the effect. | 6, Supplementary Figures S3-S14 |
| | 19b | If meta-analyses were done, present results of all investigations of possible causes of heterogeneity. | Supplementary Figures S3-S14 |
| | 19c | If meta-analyses were done, present results of all sensitivity analyses conducted to assess the robustness of synthesized results. | NA |
| Reporting biases | 20 | Present assessments (collected from systematic reviews and/or assessed anew) of the risk of bias due to missing primary studies, analyses, or results in a summary or synthesis (arising from reporting biases at the levels of the systematic reviews, primary studies, and supplemental primary studies, if included) for each summary or synthesis assessed. | NA |
| Certainty of evidence | 21 | Present assessments (collected or assessed anew) of certainty (or confidence) in the body of evidence for each outcome. | 6 |
| **DISCUSSION** | | | |
| Discussion | 22a | Summarize the main findings, including any discrepancies in findings across the included systematic reviews and supplemental primary studies (if included). | 7-8 |
| | 22b | Provide a general interpretation of the results in the context of other evidence. | 7-8 |
| | 22c | Discuss any limitations of the evidence from systematic reviews, their primary studies, and supplemental primary studies (if included) included in the overview of reviews. Discuss any limitations of the overview of reviews methods used. | 7-8 |
| | 22d | Discuss implications for practice, policy, and future research (both systematic reviews and primary research). Consider the relevance of the findings to the end users of the overview of reviews, e.g., healthcare providers, policymakers, patients, among others. | 7-8 |
| **OTHER INFORMATION** | | | |
| Registration and protocol | 23a | Provide registration information for the overview of reviews, including register name and registration number, or state that the overview of reviews was not registered. | 3 |
| | 23b | Indicate where the overview of reviews protocol can be accessed, or state that a protocol was not prepared. | NA |
| | 23c | Describe and explain any amendments to information provided at registration or in the protocol. Indicate the stage of the overview of reviews at which amendments were made. | NA |

| | | | |
|---|---|---|---|
| Support | 24 | Describe sources of financial or non-financial support for the overview of reviews, and the role of the funders or sponsors in the overview of reviews. | 9 |
| Competing interests | 25 | Declare any competing interests of the overview of reviews' authors. | 9 |
| **Section** Topic | # | Item | Location reported |
| Author information | 26a | Provide contact information for the corresponding author. | 1 |
| | 26b | Describe the contributions of individual authors and identify the guarantor of the overview of reviews. | 9 |
| Availability of data and other materials | 27 | Report which of the following are available, where they can be found, and under which conditions they may be accessed: template data collection forms; data collected from included systematic reviews and supplemental primary studies; analytic code; any other materials used in the overview of reviews. | 9 |

**Supplementary Table S2:** PRISMA checklist for Preferred Reporting Items for Systematic Reviews and Meta-Analyses

| Section and Topic | Item # | Checklist item | Location where item is reported |
|---|---|---|---|
| **TITLE** | | | |
| Title | 1 | Identify the report as a systematic review. | NA |
| **ABSTRACT** | | | |
| Abstract | 2 | See the PRISMA 2020 for Abstracts checklist. | NA |
| **INTRODUCTION** | | | |
| Rationale | 3 | Describe the rationale for the review in the context of existing knowledge. | 2 |
| Objectives | 4 | Provide an explicit statement of the objective(s) or question(s) the review addresses. | 2 |
| **METHODS** | | | |
| Eligibility criteria | 5 | Specify the inclusion and exclusion criteria for the review and how studies were grouped for the syntheses. | 3 |
| Information sources | 6 | Specify all databases, registers, websites, organisations, reference lists and other sources searched or consulted to identify studies. Specify the date when each source was last searched or consulted. | 3 |
| Search strategy | 7 | Present the full search strategies for all databases, registers and websites, including any filters and limits used. | 3, Supplementary Table S3 |
| Selection process | 8 | Specify the methods used to decide whether a study met the inclusion criteria of the review, including how many reviewers screened each record and each report retrieved, whether they worked independently, and if applicable, details of automation tools used in the process. | 3 |
| Data collection process | 9 | Specify the methods used to collect data from reports, including how many reviewers collected data from each report, whether they worked independently, any processes for obtaining or confirming data from study investigators, and if applicable, details of automation tools used in the process. | 4 |
| Data items | 10a | List and define all outcomes for which data were sought. Specify whether all results that were compatible with each outcome domain in each study were sought (e.g. for all measures, time points, analyses), and if not, the methods used to decide which results to collect. | 4 |
| | 10b | List and define all other variables for which data were sought (e.g. participant and intervention characteristics, funding sources). Describe any assumptions made about any missing or unclear information. | 4 |
| Study risk of bias assessment | 11 | Specify the methods used to assess risk of bias in the included studies, including details of the tool(s) used, how many reviewers assessed each study and whether they worked independently, and if applicable, details of automation tools used in the process. | 4 |
| Effect measures | 12 | Specify for each outcome the effect measure(s) (e.g. risk ratio, mean difference) used in the synthesis or presentation of results. | 5 |
| Synthesis methods | 13a | Describe the processes used to decide which studies were eligible for each synthesis (e.g. tabulating the study intervention characteristics and comparing against the planned groups for each synthesis (item #5)). | 5 |
| | 13b | Describe any methods required to prepare the data for presentation or synthesis, such as handling of missing summary statistics, or data conversions. | 5 |
| | 13c | Describe any methods used to tabulate or visually display results of individual studies and syntheses. | 5 |

| Section and Topic | Item # | Checklist item | Location where item is reported |
|---|---|---|---|
| | 13d | Describe any methods used to synthesize results and provide a rationale for the choice(s). If meta-analysis was performed, describe the model(s), method(s) to identify the presence and extent of statistical heterogeneity, and software package(s) used. | 5 |
| | 13e | Describe any methods used to explore possible causes of heterogeneity among study results (e.g. subgroup analysis, meta-regression). | NA |
| | 13f | Describe any sensitivity analyses conducted to assess robustness of the synthesized results. | NA |
| Reporting bias assessment | 14 | Describe any methods used to assess risk of bias due to missing results in a synthesis (arising from reporting biases). | NA |
| Certainty assessment | 15 | Describe any methods used to assess certainty (or confidence) in the body of evidence for an outcome. | 5 |
| **RESULTS** | | | |
| Study selection | 16a | Describe the results of the search and selection process, from the number of records identified in the search to the number of studies included in the review, ideally using a flow diagram. | 5 |
| | 16b | Cite studies that might appear to meet the inclusion criteria, but which were excluded, and explain why they were excluded. | - |
| Study characteristics | 17 | Cite each included study and present its characteristics. | Table 2 |
| Risk of bias in studies | 18 | Present assessments of risk of bias for each included study. | 5-6, Figure 1 |
| Results of individual studies | 19 | For all outcomes, present, for each study: (a) summary statistics for each group (where appropriate) and (b) an effect estimate and its precision (e.g. confidence/credible interval), ideally using structured tables or plots. | 6, Supplementary Figures S3-S14 |
| Results of syntheses | 20a | For each synthesis, briefly summarise the characteristics and risk of bias among contributing studies. | 6 |
| | 20b | Present results of all statistical syntheses conducted. If meta-analysis was done, present for each the summary estimate and its precision (e.g. confidence/credible interval) and measures of statistical heterogeneity. If comparing groups, describe the direction of the effect. | 6, Supplementary Figures S3-S14 |
| | 20c | Present results of all investigations of possible causes of heterogeneity among study results. | NA |
| | 20d | Present results of all sensitivity analyses conducted to assess the robustness of the synthesized results. | NA |
| Reporting biases | 21 | Present assessments of risk of bias due to missing results (arising from reporting biases) for each synthesis assessed. | NA |
| Certainty of evidence | 22 | Present assessments of certainty (or confidence) in the body of evidence for each outcome assessed. | 6 |
| **DISCUSSION** | | | |
| Discussion | 23a | Provide a general interpretation of the results in the context of other evidence. | 7-8 |
| | 23b | Discuss any limitations of the evidence included in the review. | 7-8 |

| Section and Topic | Item # | Checklist item | Location where item is reported |
|---|---|---|---|
| | 23c | Discuss any limitations of the review processes used. | 7-8 |
| | 23d | Discuss implications of the results for practice, policy, and future research. | 7-8 |
| **OTHER INFORMATION** | | | |
| Registration and protocol | 24a | Provide registration information for the review, including register name and registration number, or state that the review was not registered. | 3 |
| | 24b | Indicate where the review protocol can be accessed, or state that a protocol was not prepared. | NA |
| | 24c | Describe and explain any amendments to information provided at registration or in the protocol. | NA |
| Support | 25 | Describe sources of financial or non-financial support for the review, and the role of the funders or sponsors in the review. | 9 |
| Competing interests | 26 | Declare any competing interests of review authors. | 9 |
| Availability of data, code and other materials | 27 | Report which of the following are publicly available and where they can be found: template data collection forms; data extracted from included studies; data used for all analyses; analytic code; any other materials used in the review. | 9 |

**Supplementary Table S3**: Search strategy for identifying systematic reviews and/or randomized controlled trials in the PubMed database

| Search | Query | Results* |
|---|---|---|
| #1 | Enteral nutrition [MeSH] | 22,155 |
| #2 | Enteral feed*[Text Word] | 7,725 |
| #3 | Enteral feeding[MeSH Terms] | 22,155 |
| #4 | Enteral feeding tube[Text Word] | 272 |
| #5 | Tube feeding[Text Word] | 4,333 |
| #6 | Tube feeding[MeSH Terms] | 22,155 |
| #7 | Artificial feed*[Text Word] | 1,355 |
| #8 | Artificial nutrition[Text Word] | 1,424 |
| #9 | Gastric feed*[Text Word] | 419 |
| #10 | Intragastric feed*[Text Word] | 229 |
| #11 | Tube feed[Text Word] | 109 |
| #12 | Enteral formula[Text Word] | 506 |
| #13 | Nutritional support[Text Word] | 15,402 |
| #14 | Nutrition support[Text Word] | 3,706 |
| #15 | Parenteral nutrition[MeSH Terms] | 25,514 |
| #16 | Parenteral feed*[Text Word] | 1,718 |
| #17 | Parenteral feeding[MeSH Terms] | 25,514 |
| #18 | Parenteral formula[Text Word] | 12 |
| #19 | Parenteral tube feeding[Text Word] | 521 |
| #20 | PEG line[Text Word] | 7,395 |
| #21 | EN[Text Word] | 77,674 |
| #22 | PN[Text Word] | 26,468 |
| #23 | Supplemental parenteral nutrition | 172 |

| | | |
|---|---|---|
| | [Text Word] | |
| #24 | Artificial supplementation[Text Word] | 21 |
| #25 | Supplemental enteral nutrition[Text Word] | 21 |
| #26 | Intravenous supplementation[Text Word] | 174 |
| #27 | 1 OR 2 OR 3 OR 4 OR 5 OR 6 OR 7 OR 8 OR 9 OR 10 OR 11 OR 12 OR 13 OR 14 OR 15 OR 16 OR 17 OR 18 OR 19 OR 20 OR 21 OR 22 OR 23 OR 24 OR 25 OR 26 | 169,491 |
| #28 | Critical care[MeSH Terms] | 67,579 |
| #29 | Critical care[Text Word] | 91,884 |
| #30 | ICU[Text Word] | 88,582 |
| #31 | Intensive care unit[Text Word] | 137,470 |
| #32 | Intensive care[Text Word] | 237,182 |
| #33 | Critical ill*[Text Word] | 47,764 |
| #34 | Critical illness[MeSH Terms] | 39,835 |
| #35 | Shock[MeSH Terms] | 87,052 |
| #36 | Shock[Text Word] | 265,017 |
| #37 | Sepsis[Text Word] | 154,505 |
| #38 | Sepsis[MeSH Terms] | 144,031 |
| #39 | Multiple organ failure[MeSH Terms] | 12,159 |
| #40 | Multiple organ failure*[Text Word] | 18,266 |
| #41 | Systemic inflammatory response syndrome [MeSH Terms] | 152,095 |
| #42 | Systemic inflammatory response syndrome [Text Word] | 11,348 |
| #43 | Mechanical ventilation[MeSH Terms] | 90,022 |
| #44 | Mechanical ventilat*[Text Word] | 62,953 |

| | | |
|---|---|---|
| #45 | 28 OR 29 OR 30 OR 31 OR 32 OR 33 OR 34 OR 35 OR 36 OR 37 OR 38 OR 39 OR 40 OR 41 OR 42 OR 43 or 44 | 814,598 |
| #46 | Systematic review[Text Word] | 330,197 |
| #47 | Systematic review[Publication Type] | 250,139 |
| #48 | Review[Publication Type] | 3,273,819 |
| #49 | Review[Text Word] | 4,128,298 |
| #50 | Meta-analysis[Text Word] | 295,382 |
| #51 | Meta-analysis[Publication Type] | 193,465 |
| #52 | 46 OR 47 OR 48 OR 49 OR 50 OR 51 | 4,222,293 |
| #53 | #27 AND #45 AND #52 | 4,024 |
| #54 | #27 AND #45 AND #52 Replacing Text Word to Title/Abstract | 3,671 |
| #55 | #27 AND #45 AND #52 Replacing Text Word to Title/Abstract Filters: from 2000-2024 | 2,964 |
| #56 | Randomized[Title/Abstract] | 690,630 |
| #57 | Random[Title/Abstract] | 369,340 |
| #58 | Randomly[Title/Abstract] | 426,287 |
| #59 | Randomized controlled trial[Title/Abstract] | 117,069 |
| #60 | Randomized controlled trial [Publication Type] | 608,622 |
| #61 | Randomised controlled trial[Title/Abstract] | 32,931 |
| #62 | Randomized trial[Title/Abstract] | 55,422 |
| #63 | Randomised trial[Title/Abstract] | 11,542 |
| #64 | Randomized clinical trial[Title/Abstract] | 41,775 |
| #65 | Randomised clinical trial[Title/Abstract] | 4,518 |
| #66 | Controlled clinical trial[Title/Abstract] | 20,085 |
| #67 | Trial[Title/Abstract] | 801,558 |

| | | |
|---|---|---|
| #68 | 56 OR 57 OR 58 OR 59 OR 60 OR 61 OR 62 OR 63 OR 64 OR 65 OR 66 OR 67 | 1,901,947 |
| #69 | #27 AND #45 AND #68 | 2,480 |

**Supplementary Table S4**: Detailed reasons for exclusion of the systematic reviews

| Number | Title | Author | Type of intervention/control-Reason of exclusion |
|---|---|---|---|
| 1 | Small peptide formulas versus standard polymeric formulas in critically ill patients with acute gastrointestinal injury: a systematic review and meta-analysis | Wang Y et al. | Intervention: small peptide formula EN<br>Comparison: standard polymeric formula EN |
| 2 | Effect of Adopting Low Calories on Patients' Vital Signs in The Nutritional Support of Critically Ill Patients in the ICU: A Systematic Review and Network Meta-Analysis | Jiang Q, Duan L. et al. | Intervention: low caloric intake with no PN supplementation |
| 3 | A systematic review of the safety and tolerability evaluation of enteral nutrition in a prone position with acute respiratory distress syndrome | Liu Y. et al. | Intervention and comparison EN according to patient position |
| 4 | Timing of enteral nutrition and parenteral nutrition in the PICU | Fell DM. et al. | Referred to pediatric population-study EN vs EN+PN |
| 5 | The effect of intermittent versus continuous enteral feeding for critically ill patients: a meta-analysis of randomized controlled trials | Qu J. et al. | I: Intermittent EN<br>C: Continuous EN |
| 6 | Nutritive Support for Critical Exotic Patients | Latney LV. et al. | P: Animals |
| 7 | Enteral feeding tolerance during pharmacologic neuromuscular blockade | Dickerson RN. et al. | Intervention and control include EN |
| 8 | Blood glucose monitoring in critically ill adult patients: type of sample and method of analysis. Systematic review and meta-analysis | Arias-Rivera S. et al. | It measures blood glucose in ICU patients but it does not refer to the nutrition therapy type they receive-not in English |
| 9 | Lipid emulsions in parenteral nutrition: does it matter? | Haines K. et al. | Intervention and comparison include PN |
| 10 | Optimizing enteral nutrition delivery by implementing volume-based feeding protocol for critically ill patients: an updated meta-analysis and systematic review | Wang L. et al. | Intervention and comparison include EN |
| 11 | Efficacy of enteral nutrition for patients with acute pancreatitis: A systematic review and meta-analysis of 17 studies | Liu Y. et al. | Intervention and comparison include EN |
| 12 | Early vs delayed enteral nutrition or parenteral nutrition in hospitalized patients: An umbrella review of systematic reviews and meta-analyses of randomized trials | Talebi S. et al. | I: EEN<br>C:DEN or PN |
| 13 | Prophylactic acid suppression and enteral nutrition | Barletta JF. et al. | I: PPI and EN<br>C: EN |

| | | | |
|---|---|---|---|
| 14 | Omega-3 fatty acids in parenteral nutrition - A systematic review with network meta-analysis on clinical outcomes | Pradelli L. et al. | I: Ω3 in PN<br>C: PN |
| 15 | [Immunomodulation by nutritional intervention in critically ill patients] | Hirschberger S. et al. | In German |
| 16 | Efficacy and safety of early enteral and intravenous fluid resuscitation in severe acute pancreatitis: a systematic review and meta-analysis | Liu J. et al. | I: EN<br>C:iv fluids |
| 17 | Comparison of continuous versus intermittent enteral feeding in critically ill patients: a systematic review and meta-analysis | Heffernan AJ. et al. | I: Intermittent EN<br>C: Continuous EN |
| 18 | Effects of dietary fiber on enteral feeding intolerance and clinical outcomes in critically ill patients: A meta-analysis | Liu T. et al. | I: Fiber enriched EN<br>C: EN standard |
| 19 | Administration of vitamin D and its metabolites in critically ill adult patients: an updated systematic review with meta-analysis of randomized controlled trials | Menger J. et al. | $I_1$: Intervention group EN enriched with Vit D<br>$C_1$: EN standard<br>$I_2$:PN enriched Vit D<br>$C_2$: PN standard |
| 20 | Higher versus lower enteral calorie delivery and gastrointestinal dysfunction in critical illness: A systematic review and meta-analysis | Murthy TA. et al. | I: High caloric EN<br>C: Low caloric EN |
| 21 | Effect of Early Low-Calorie Enteral Nutrition Support in Critically Ill Patients: A Systematic Review and Meta-analysis | Jiang Q. et al. | Intervention and control refer to EN |
| 22 | A systematic review on the agreement between clinical practice guidelines regarding the steps of the nutrition care process of adult patients who are critically ill | Cattani A. et al. | There is no comparison to given intervention |
| 23 | Challenges to Provision of Adequate Medical Nutrition Therapy in a Critically Ill COVID-19 Patient Fed in the Prone Position | Brown T. et al. | Narrative review about EN |

| | | | |
|---|---|---|---|
| 24 | Effect of Early Nutritional Support on Clinical Outcomes of Critically Ill Patients with Sepsis and Septic Shock: A Single-Center Retrospective Study | Cha JK. et al. | Retrospective study |
| 25 | Effect of enteral immunonutrition compared with enteral nutrition on surgical wound infection, immune and inflammatory factors, serum proteins, and cellular immunity in subjects with gastric cancer undergoing a total gastrectomy: A meta-analysis | Fu H. et al. | I: EN<br>C:EN immunonutrition |
| 26 | Clinical Outcomes of Zinc Supplementation Among COVID-19 Patients | Berti AD. et al. | Non ICU and intervention and control receive only EN with Zinc supplementation |
| 27 | The Effects of Enteral Nutrition in Critically Ill Patients with COVID-19: A Systematic Review and Meta-Analysis | Ojo O. et al. | $I_1$: EEN<br>$C_1$: DEN<br>$I_2$: EN<br>$C_2$:PN |
| 28 | Omega-6 sparing effects of parenteral lipid emulsions-an updated systematic review and meta-analysis on clinical outcomes in critically ill patients | Notz Q. et al. | I:PN enriched fish oil<br>C: PN standard |
| 29 | Safety and efficacy of continuous or intermittent enteral nutrition in patients in the intensive care unit: Systematic review of clinical evidence | De Lazzaro F. et al. | I: Intermittent EN<br>C: Continuous EN |
| 30 | The Protective Role of Nutraceuticals in Critically Ill Patients with Traumatic Brain Injury | Rahimibashar F. et al. | Refer to EN |
| 31 | Systematic review and meta-analysis of the effectiveness of continuous vs intermittent enteral nutrition in critically ill adults | Thong D. et al. | I: intermittent EN<br>C: continuous EN in ICU |
| 32 | A systematic review and meta-analysis of the effect of total parenteral nutrition and enteral nutrition on the prognosis of patients with acute pancreatitis | Liu M. et al. | I: TPN<br>C:TEN |
| 33 | Micronutrient intake from enteral nutrition in critically ill adults: A systematic review of randomised controlled trials | Breik L. et al. | Intervention and control refer to EN |
| 34 | Efficacy of volume-based feeding (VBF) protocol on critically ill patients: A meta-analysis and systematic review | Wang L. et al. | I: EN VBF<br>C: RBF VBF* |

| | | | |
|---|---|---|---|
| | | | * (volume based feeding) |
| 35 | Glutamine as a component of nutritional and metabolic therapy for surgical patients in ICU | Dmitriev AV. et al. | In Russian |
| 36 | Fiber in the ICU: Should it Be a Regular Part of Feeding? | Green CH. et al. | I: EN enriched with fiber<br>C: EN standard |
| 37 | Effect of enteral immunomodulatory nutrition formula on mortality and critical care parameters in critically ill patients: A systematic review with meta-analysis | Malekahmadi M. et al. | I: EN immunomodulatory formula<br>C:EN standard |
| 38 | Effect of glutamine supplementation on inflammatory markers in critically ill patients supported with enteral or parenteral feeding | Gholamalizadeh M. et al. | I: EN with glutamine<br>C:PN with Glutamine |
| 39 | The effect of standardized feeding protocol on early outcome following gastroschisis repair: A systematic review and meta-analysis | Raduma OS. et al. | I: EN<br>C:PN |
| 40 | Nutritional Interventions on Physical Functioning for Critically Ill Patients: An Integrative Review | Zhou W. et al. | I: Early EN<br>C:Early PN |
| 41 | Safety of Using Enteral Nutrition Formulations Containing Dietary Fiber in Hospitalized Critical Care Patients: A Systematic Review and Meta-Analysis | Cara KC. et al. | Intervention and control receive EN |
| 42 | Association of specialized enteral nutrition with glycemic control and clinical outcomes in critically ill patients: Meta-analysis of randomized controlled trials | Eckert I. et al. | Intervention and control receive EN |
| 43 | Diet Recommendations for Hospitalized Patients With Inflammatory Bowel Disease: Better Options Than Nil Per Os | Palchaudhuri S. et al. | P: non-ICU patients |
| 44 | Feeding intolerance in critically ill patients with COVID-19 | Liu R. et al. | Intervention and control receive EN |
| 45 | REPORT-Parenteral selenium for the clinical effect of sepsis: A meta-analysis of randomized controlled trials | He S. et al. | I: IV selenium<br>C:placebo does not refer to enteral |
| 46 | [Nutrition support in the chronic critically ill patients] | Wang L. et al. | Narrative review |

| | | | |
|---|---|---|---|
| 47 | Low-carbohydrate, high-fat enteral formulas for managing glycemic control in patients who are critically ill: A review of the evidence | Burslem R. et al. | I: Low carbohydrate EN<br><br>C:High fat EN content |
| 48 | Efficacy of alanyl glutamine in nutritional support therapy for patients with sepsis: A protocol for systematic review and meta-analysis | Su X. et al. | I: EN with alanyl glutamine<br><br>C: PN with alanyl glutamine |
| 49 | Intravenous hyperalimentation: a review | Freeman JB. et al. | Not found |
| 50 | Nutritional care of the critically ill patient with respiratory failure | Weissman C. et al. | Not found |
| 51 | The critically ill surgical patient: nutritional considerations | Starker PM. et al. | Not found |
| 52 | The patient with critical neurological disease | Young B. et al. | Not found |
| 53 | Nutritional support in the management of critically ill patients in surgical intensive care | Streat SJ. et al. | Not found |
| 54 | Parenteral and enteral nutrition and the enterocutaneous fistula treatment. II. Factors influencing the outcomes of treatment | Dardai E. et al. | I: TPN<br><br>C:EN |
| 55 | Parenteral and enteral nutrition | Shizgal et al. | I: EN<br><br>C:PN |
| 56 | Special considerations required for the formulation and administration of total parenteral nutrition therapy in the elderly patient | Driscoll DF. et al. | TPN in non-ICU |
| 57 | Should we supplement magnesium in critically ill patients? | Olerich MA. et al. | Article cannot be found |
| 58 | Omega-3 polyunsaturated fatty acids: benefit or harm during sepsis? | Peck. et al. | Article cannot be found |
| 59 | Growth hormone administration during nutritional support: what is to be gained? | Ziegler TR. et al. | Article cannot be found |
| 60 | Does the formulation of enteral feeding products influence infectious morbidity and mortality rates in the critically ill patients? A critical review of the evidence | Heyland DK. et al. | I: Formulated EN<br><br>C: EN standard |
| 61 | [Nutrition physiologic significance of the trace element selenium within the scope of parenteral nutrition therapy] | Gramm HJ. et al. | Article not found |

| | | | |
|---|---|---|---|
| 62 | The necessity of selenium substitution in total parenteral nutrition and artificial alimentation | Gramm HJ. et al. | I: TPN with selenium<br><br>C: EN |
| 63 | Glutamine supplemented nutrition in clinical practice--use of glutamine-containing dipeptides | Fürst P. et al. | I: Glutamine EN<br><br>C: Glutamine PN |
| 64 | Parenteral glutamine supply in intensive care patients | R D Griffiths . et al. | I:PN with glutamine<br><br>C:PN standard |
| 65 | Enteral nutritional supplementation with key nutrients in patients with critical illness and cancer: a meta-analysis of randomized controlled clinical trials | Heys SD. et al. | I: EN supplemented<br><br>C: EN standard |
| 66 | Glutamine-containing TPN: a question of life and death for intensive care unit-patients? | Wernerman J. et al. | I: TPN with glutamine<br><br>C: TPN |
| 67 | Medium-chain triglyceride and n-3 polyunsaturated fatty acid-containing emulsions in intravenous nutrition | Chan S. et al. | I: MCT PN<br><br>C: PUFA PN |
| 68 | Prioritizing nutrition during recovery from critical illness | Nienow MK. et al. | EN and oral supplements |
| 69 | Effects of intermittent feeding versus continuous feeding on enteral nutrition tolerance in critically ill patients: A protocol for systematic review and meta-analysis | Li Y. et al. | I: Intermittent EN<br><br>C: Continuous EN |
| 70 | [Key problems of nutritional support in patients with ischemic stroke and nontraumatic intracranial hemorrhage] | Khoshbonyani PA. et al. | Only abstract in English was detectable |
| 71 | How to Feed the Critically Ill-A Review | Lew CC. et al. | Narrative review |
| 72 | Intermittent versus continuous enteral nutrition on feeding intolerance in critically ill adults: A meta-analysis of randomized controlled trials | Ma Y. et al. | I: Intermittent EN<br><br>C: Continuous EN |
| 73 | Omega-3 fatty acid-containing parenteral nutrition in ICU patients: systematic review with meta-analysis and cost-effectiveness analysis | Pradelli L. et al. | I: Standard PN<br><br>C: PN supplemented with Ω3 FA |

| | | | |
|---|---|---|---|
| 74 | Reporting of Randomized Controlled Trials Investigating an Enteral or Parenteral Nutrition Intervention in Critical Illness According to the CONSORT Statement: A Systematic Review and Recommendation of Minimum Standard Reporting Criteria | Seddon N. et al. | This SR and MA does not study the wanted topic but evaluates the published RCTs about nutrition in ICU according to CONSORT criteria |
| 75 | Early Achievement of Enteral Nutrition Protein Goals by Intensive Care Unit Day 4 is Associated With Fewer Complications in Critically Injured Adults | Hartwell JL. et al. | Study about EN and energy requirements goals |
| 76 | Nutritional support for critically ill patients suffering from SARS-CoV-2 infection | Pardo E. et al. | Not in English |
| 77 | Meta-analysis of the effects of supplemental parenteral nutrition on prognosis of critically ill patients | Chu X. et al. | Not in English |
| 78 | Challenges of Maintaining Optimal Nutrition Status in COVID-19 Patients in Intensive Care Settings | Minnelli N. et al. | Narrative review |
| 79 | An evaluation of feeding practices and determination of barriers to providing nutritional support in a multidisciplinary South African intensive care unit | Elmezoughi E. et al. | Retrospective observational chart review |
| 80 | Nutrition Support in Critically Ill Patients with AKI | Ramakrishnan N. et al. | Narrative review of nutrition in ICU |
| 81 | Nutrition Support in the ICU-A Refresher in the Era of COVID-19 | Micic D. et al. | Narrative review |
| 82 | The clinical and cost-effectiveness of supplemental parenteral nutrition in oncology | Webb N. et al. | Narrative review of SPN in cancer patients |
| 83 | Continuous versus intermittent feeding of the critically ill: have we made progress? | Pletschette Z. et al. | I: Intermittent EN<br><br>C: Continuous EN |
| 84 | Therapeutic Effect of Enteral Nutrition Supplemented with Probiotics in the Treatment of Severe Craniocerebral Injury: A Systematic Review and Meta-Analysis | Du T. et al. | I: EN supplemented with probiotics<br><br>C: EN standard, does not specify the way of supplementation |
| 85 | Effects of parenteral glutamine in critically ill surgical patients: a systematic review and meta-analysis | Pimentel RFW. et al. | Not in English (Spanish) |
| 86 | Meta-Analysis of Efficacy of Rhubarb Combined With Early Enteral Nutrition for the Treatment of Severe Acute Pancreatitis | Chen X. et al. | I: EN<br><br>C: EN with Rhubarb |
| 87 | Outcome Measures in Critical Care Nutrition Interventional Trials: A Systematic Review | Chapple LS. et al. | It studies general nutritional interventions |
| 88 | Nutritional support for critically ill patients in the intensive care unit | Hagve M. et al. | Not in English |

| | | | |
|---|---|---|---|
| 89 | When is parenteral nutrition indicated in the hospitalized, acutely ill patient? | Fragkos KC. et al. | Narrative review for PN |
| 90 | Immunonutrition vs Standard Nutrition for Cancer Patients: A Systematic Review and Meta-Analysis (Part 1) | Yu K. et al. | Immunonutrition vs standard nutrition in cancer patients not in ICU |
| 91 | Impact of Soluble Fiber in the Microbiome and Outcomes in Critically Ill Patients | Venegas-Borsellino C. et al. | Narrative review |
| 92 | Systematic Review With Meta-Analysis of Patient-Centered Outcomes, Comparing International Guideline-Recommended Enteral Protein Delivery With Usual Care | Fetterplace K. et al. | I: EN guidelines<br><br>C: Standard care |
| 93 | Immunonutrition for Adults With ARDS: Results From a Cochrane Systematic Review and Meta-Analysis | Ahilanandan Dushianthan. et al. | Given enteral or parenteral immunonutrients, additionally supplemented with or as part of a nutritional formula. In comparison, control groups included participants who received placebo or standard nutrition with a non-immunonutrient formula feed. |
| 94 | Medical Nutrition Therapy in Critically Ill Patients Treated on Intensive and Intermediate Care Units: A Literature Review | Kopp Lugli A. et al. | Literature review |
| 95 | Effects of Early Enteral Glutamine Supplementation on Intestinal Permeability in Critically Ill Patients | Shariatpanahi ZV. et al. | I: EEN with glutamine<br><br>C: EN standard |
| 96 | High-Carbohydrate vs High-Fat Nutrition for Burn Patients | Shields BA. et al. | P:in burned patients<br><br>I: EN<br><br>C: PN |
| 97 | Early enteral nutrition within 24 hours of lower gastrointestinal surgery versus later commencement for length of hospital stay and postoperative complications | Herbert G. et al. | I: EEN<br><br>C: DEN |
| 98 | Enteral vs. parenteral nutrition in septic shock: are they equivalent? | Kott M. et al. | I: EN<br><br>C:PN |
| 99 | Intermittent or continuous feeding: any difference during the first week? | Van Dyck L. et al. | I: Intermittent EN<br><br>C: Continuous EN |

| | | | |
|---|---|---|---|
| 100 | The Benefits of Parenteral Nutrition (PN) Versus Enteral Nutrition (EN) Among Adult Critically Ill Patients: What is the Evidence? A Literature Review | Cadena AJ. et al. | I:PN<br>C: EN |
| 101 | Early enteral nutrition supplemented with probiotics improved the clinical outcomes in severe head injury: Some promising findings from Chinese patients | Yi LJ. et al. | I: EEN with probiotics<br>C: EN standard |
| 102 | Omega-3 polyunsaturated fatty acids in critically ill patients with acute respiratory distress syndrome: A systematic review and meta-analysis | Langlois PL. et al. | I: EN with Ω3 fatty acids<br>C: EN |
| 103 | Impact of Intravenous Lipid Emulsions Containing Fish Oil on Clinical Outcomes in Critically Ill Surgical Patients: A Literature Review | Honeywell S. et al. | Narrative review |
| 104 | Current evidence on ω-3 fatty acids in enteral nutrition in the critically ill: A systematic review and meta-analysis | Kristine Koekkoek W. et al. | I: EN with Ω3 FA<br>C: EN standard |
| 105 | What is the evidence for the use of parenteral nutrition (PN) in critically ill surgical patients: a systematic review and meta-analysis | Ledgard K. et al. | I:PN with glutamine<br>C:PN standard |
| 106 | Vitamin C supplementation in the critically ill: A systematic review and meta-analysis | Zhang M. et al. | Intravenous vitamin C supplementation versus placebo or no intervention, with no EN provision. |
| 107 | Efficacy comparisons of enteral nutrition and parenteral nutrition in patients with severe acute pancreatitis: a meta-analysis from randomized controlled trials | Wu P. et al. | I: EN<br>C:PN |
| 108 | Use of dietary fibers in enteral nutrition of critically ill patients: a systematic review | Reis AMD. et al. | I: EN enriched with fibers<br>C: EN standard, narrative review |
| 109 | Early Enteral Nutrition Reduces Mortality and Improves Other Key Outcomes in Patients With Major Burn Injury: A Meta-Analysis of Randomized Controlled Trials | Pu H. et al. | I: EEN<br>C: DEN |
| 110 | Enteral nutrition provided within 48 hours after admission in severe acute pancreatitis: A systematic review and meta-analysis | Song J. et al. | I: EEN<br>C: DEN |
| 111 | The effect of enteral versus parenteral nutrition for critically ill patients: A systematic review and meta-analysis | Zhang G. et al. | I: EN<br>C:PN |

| | | | |
|---|---|---|---|
| 112 | Supplemental Parenteral Nutrition: Review of the Literature and Current Nutrition Guidelines | Russell MK. et al. | Literature review of guidelines |
| 113 | Early enteral nutrition versus delayed enteral nutrition in acute pancreatitis: A PRISMA-compliant systematic review and meta-analysis | Feng P. et al. | I: EEN<br>C: DEN |
| 114 | Initial energy supplementation in critically ill patients receiving enteral nutrition: a systematic review and meta-analysis of randomized controlled trials | Tian F. et al. | I: Low energy EN<br>C: High energy EN |
| 115 | Effects of perioperative supplementation with pro-/synbiotics on clinical outcomes in surgical patients: A meta-analysis with trial sequential analysis of randomized controlled trials | Wu XD. et al. | I: EN supplementation with pro-/synbiotics<br>C: Standard EN |
| 116 | Efficacy of glutamine-enriched enteral feeding formulae in critically ill patients: a systematic review and meta-analysis of randomized controlled trials | Mottaghi A. et al. | I: glutamine-enriched enteral feeding<br>C: EN standard |
| 117 | Cost and effectiveness of omega-3 fatty acid supplementation in Chinese ICU patients receiving parenteral nutrition | Wu GH. et al. | I: omega-3 fatty acid supplementation PN<br>C: Standard PN |
| 118 | The role of ω-3 fatty acid supplemented parenteral nutrition in critical illness in adults: a systematic review and meta-analysis | Palmer AJ. et al. | I: ω-3 fatty acid supplemented parenteral nutrition<br>C: Standard parenteral nutrition |
| 119 | n-3 fatty acid-enriched parenteral nutrition regimens in elective surgical and ICU patients: a meta-analysis | Pradelli L. et al. | I: n-3 fatty acid-enriched parenteral nutrition<br>C: Standard PN |
| 120 | Combined enteral and parenteral nutrition | Wernerman J. et al. | Systematic review |
| 121 | n-3 PUFAs in cancer, surgery, and critical care: a systematic review on clinical effects, incorporation, and washout of oral or enteral compared with parenteral supplementation | Langius JA. et al. | $I_1$: n-3 PUFAs EN<br>$I_2$: n-3 PUFAs PN<br>C: oral feeding |
| 122 | Zinc supplementation in critically ill patients: a key pharmaconutrient? | Heyland DK. et al. | I: Zinc iv<br>C: Zinc in EN |

| | | | |
|---|---|---|---|
| 123 | Enteral and parenteral nutrition in the seriously ill, hospitalized patient: a critical review of the evidence | Heyland DK. et al. | Narrative review |
| 124 | Nutritional supplementation in elderly people during the course of catabolic illnesses | Bourdel-Marchasson I. et al. | Narrative review |
| 125 | Nutrition in Acute Pancreatitis: From the Old Paradigm to the<br><br>New Evidence | Lucia et al. | Narrative review about nutrition in acute pancreatitis |
| 126 | Systematic review and meta-analysis of the effect of nutritional support on the clinical outcome of patients with traumatic brain injury | Yang L. et al. | P: adult population in ICU with traumatic brain injury and GCS 3-8<br><br>I: Enriched EN (in the SR it is referred as intensive nutrition-all the included RCTs revised in order to specify the term)<br><br>C: EN<br><br>O: Mortality, alterations in immune status, energy intake |
| 127 | Burn Patient Metabolism and Nutrition | Nunez JH. et al. | Narrative review |
| 128 | Role of vitamin D supplementation in modifying outcomes after surgery: a systematic review of randomised controlled trials | Patel A. et al. | P: Pediatric and adult patients<br><br>I: VitD supplementation<br><br>C: no supplementation and it |
| 129 | Effect of combined parenteral and enteral nutrition for patients with a critical illness: A meta-analysis of randomized controlled trials | Luo Y. et al. | P: adults and children with critical illness in ICU and other departments<br><br>I: EN and PN<br><br>C: EN<br><br>O: Mortality, LOS, UTIs, Respiratory infections |
| 130 | Effect of supplemental parenteral nutrition on all-cause mortality in critically Ill adults: A meta-analysis and subgroup analysis | Li P. et al. | Retracted article |
| 131 | Early parenteral nutrition alone or accompanying enteral nutrition in critically ill patients: a systematic review and meta-analysis | Wan X. et al. | P: critically ill in ICU stayed more than 48 hours<br><br>I: PN alone in 48 hours or PN +EN |

| | | | |
|---|---|---|---|
| | | | C: unfed<br><br>O: LOS, Mortality, mechanical ventilation duration |
| 132 | The Effectiveness of Early Enteral Nutrition on Clinical Outcomes in Critically Ill Sepsis Patients: A Systematic Review | Moon SJ. et al. | I: EEN<br><br>C: DEN |
| 133 | Clinical efficacy of enteral nutrition feeding modalities in critically ill patients: a systematic review and meta-analysis of randomized controlled trials | Wu JY. et al. | I: Intermittent EN<br><br>C: continuous EN |
| 134 | The Efficacy of Parenteral Nutrition and Enteral Nutrition Supports in Traumatic Brain Injury: A Systemic Review and Network Meta-Analysis | Qin Y. et al. | Compare EN and PN in burned patients not in ICU |
| 135 | The Effect of immunonutrition in patients undergoing pancreaticoduodenectomy: a systematic review and meta-analysis | Fan Y. et al. | P: Patients undergoing pancreaticoduodenectomy not specify if are hospitalized in ICU<br><br>I: Oral or EN or PN immunonutrition<br><br>C: no supplementation with immunonutrition |
| 136 | Nutritional Support with Omega-3 Fatty Acids in Burn Patients: A Systematic Review with Meta-Analysis of Randomized Controlled Trials | Siritientong T. et al. | P: Burn patients not referred in ICU<br><br>I: EN enriched Ω3 FA<br><br>C: EN standard<br><br>O: Mortality, LOS, energy intake |
| 137 | Effect of Early Versus Delayed Parenteral Nutrition on the Health Outcomes of Critically Ill Adults: A Systematic Review | Sharma SK. et al. | P: ICU adult patients<br>The review include 5 RCTs: compared EPN and LPN, one trial compared early TPN and standard care, one trial compared supplemental PN with standard care, and two trials compared TPN and supplemental PN.<br><br>O: LOS, Mortality, duration of mechanical ventilation and infections |

| | | | |
|---|---|---|---|
| 138 | Mortality in septic patients treated with vitamin C: a systematic meta-analysis | Scholz SS. et al. | P: adult patients in ICU<br>i: intravenous vitamin C treatment alone or in combination<br>with thiamine and/or hydrocortisone<br>C: Standard care or placebo treatment<br>O: Mortality |
| 139 | Impact of Intravenous Fluids and Enteral Nutrition on the Severity of Gastrointestinal Dysfunction: A Systematic Review and Meta-analysis | Asrani VM. et al. | P: ICU patients with GI dysfunction<br>I: IV fluids and EN<br>C: PN<br>O: Mortality, vomiting, LOS |
| 140 | Immunonutrition for Adults With ARDS: Results From a Cochrane Systematic Review and Meta-Analysis | Dushianthan A. et al. | P: ICU patients with ARDS<br>I: Enriched EN or enriched PN formula<br>C: EN or PN standard<br>O: Mortality |
| 141 | Early enteral nutrition (within 48 hours) versus delayed enteral nutrition (later 48 hours) with or without supplemental parenteral nutrition in critically ill adults (Cochrane Review) | Fuentes Padilla P. et al. | P: ICU adult patients<br>I: EEN+SPN<br>C: DEN+SPN<br>O: Mortality, LOS in ICU, Infections |

**Supplementary Table S5**: Detailed reasons for exclusion of the randomized controlled trials

| Number | Title | Author | Type of intervention/Control-Reason of exclusion |
|---|---|---|---|
| 1 | Effect of Early vs Late Supplemental Parenteral Nutrition in Patients Undergoing Abdominal Surgery: A Randomized Clinical Trial | Xuejin Gao. et al. | P: patients undergoing major abdominal surgery, non-ICU department<br>I: ESPN<br>C: LSPN<br>O:Incidence of nosocomial infections, days of antibiotic therapy |
| 2 | Impact of early low-calorie low-protein versus standard-calorie standard-protein feeding on outcomes of ventilated adults with shock: design and conduct of a randomised, controlled, multicenter, open-label, parallel-group trial (NUTRIREA-3 | Reignier J. et al. | P:>18yo patients in ICU under vasoactive support<br>I: Low caloric group 6kcl/kg/d via EN or PN<br>C: Standard 25 kcal/kg/d via EN or PN<br>O: Mortality ,nosocomial infections |
| 3 | Parenteral glutamine supplementation in critical illness: a systematic review | Wischmeyer PE. et al. | Systematic review |
| 4 | Timing of parenteral nutrition in ICU patients: A transatlantic controversy. | Veraar C. et al. | Not RCT, comparator of ASPEN and ESPEN Guidelines about the time of initiation of PN |
| 5 | Effect of glutamine supplementation on inflammatory markers in critically ill patients supported with enteral or parenteral feeding | Gholamalizadeh M. et al. | Systematic review and meta-analysis referring to selenium supplementation in EN vs PN |
| 6 | Glutamine supplementation in critical illness: evidence, recommendations, and implications for clinical practice in burn care. | Windle EM. et al. | Narrative review |
| 7 | Intravenous amino acids may mediate the adverse effect of early parenteral nutrition on mortality in critically ill patients requiring mechanical ventilation: A post hoc analysis of the NEED trial. | Lin J. et al. | Post hoc analysis<br>P: ICU adult patients<br>I: EPN |

| | | | |
|---|---|---|---|
| | | | C: LPN<br><br>O:Mortality, ICU free days to 28$^{th}$ day |
| 8 | A more physiological feeding process in ICU: Intermittent infusion with semi-solid nutrients (CONSORT-compliant). | Lu K. et al. | P: ICU adult patients<br><br>I: EN intermittent feeding with semi solid nutrients<br><br>C: Intermittent feeding<br><br>O: nutritional intake, gastrointestinal disorders |
| 9 | Enteral and supplemental parenteral nutrition enriched with omega-3 polyunsaturated fatty acids in intensive care patients - A randomized, controlled, double-blind clinical trial. | Singer P. et al. | P: ICU adult patients under mechanical ventilation<br><br>I: EN+PN +Ω3<br><br>C: EN+PN<br><br>O:Mortality, change of PaO2/FiO2 from day 1 to 4 |
| 10 | The efficacy of parenteral fish oil in critical illness patients with sepsis: a prospective, non-randomized, observational study. | Li WS. et al. | P: ICU adult patients with sepsis<br><br>I: TPN +fish oil<br><br>C: TPN standard<br><br>O:Mortality, days of hospitalization |
| 11 | Four-oil intravenous lipid emulsion effect on plasma fatty acid composition, inflammatory markers and clinical outcomes in acutely ill patients: A randomised control trial (Foil fact). | Donoghue V. et al. | P: ICU adult patients<br><br>I: PN four-oil<br><br>C:PN with soybean oil<br><br>O: biomarkers, mortality, days of hospitalization |
| 12 | Efficacy and Safety of Glutamine-supplemented Parenteral Nutrition in Surgical ICU Patients: An American Multicenter Randomized Controlled Trial. | Ziegler TR. et al. | P: ICU adult patients after cardiovascular or gastrointestinal surgery<br><br>I: PN glutamine supplemented<br><br>C: PN standard<br><br>O: Mortality, days of hospitalization |

| | | | |
|---|---|---|---|
| 13 | A randomized controlled trial investigating the effects of parenteral fish oil on survival outcomes in critically ill patients with sepsis: a pilot study. | Hall TC. et al. | P: ICU adult patients with sepsis<br><br>I:PN enriched with fish oil<br><br>C: standard care with antibiotics and iv fluids<br><br>O:inflammatory markers, mortality |
| 14 | Early enteral and parenteral nutritional support in patients with cirrhotic portal hypertension after pericardial devascularization. | Zhang K. et al. | C: ICU adult patients after pericardial devascularization<br><br>I: EN<br><br>C:PN<br><br>O: mortality, complication rate, stay in ICU, duration of hospitalization and costs of treatment |
| 15 | Infection, multiple organ failure, and survival in the intensive care unit: influence of glutamine-supplemented parenteral nutrition on acquired infection. | Griffiths RD. et al. | P: ICU adult patients<br><br>I: TPN glutamine-supplemented<br><br>C: TPN isonitrogenous, isoenergetic<br><br>O: Nosocomial infection |
| 16 | Evaluating the significance of delaying intravenous lipid therapy during the first week of hospitalization in the intensive care unit",", Lemon S." | Arrazcaeta J. et al. | P: ICU trauma adult patients<br><br>I: EPN enriched with lipids earlier than 7$^{th}$ day<br><br>P: LPN enriched with lipids later than 7$^{th}$ day<br><br>O:Mortality, days of ICU stay |
| 17 | Early enteral supplementation with key pharmaconutrients improves Sequential Organ Failure Assessment score in critically ill patients with sepsis: outcome of a randomized, controlled, double-blind trial. | Beale RJ. et al. | P: ICU adult patients with sepsis<br><br>I: EN supplemented pharmaconutrients<br><br>C: EN standard<br><br>O: Mortality, nosocomial infections, days of hospitalization |
| 18 | Comparative study on the enteral and parenteral nutrition during early postburn stage in burn patients. | Chen ZY. et al. | In Chinese |

| | | | |
|---|---|---|---|
| 19 | One year outcomes in patients with acute lung injury randomised to initial trophic or full enteral feeding: prospective follow-up of EDEN randomised trial. | Needham DM. et al. | P: patients with lung disease in non-ICU department<br><br>I: per os feeding<br><br>C: EN via tube<br><br>O: mortality |
| 20 | Fish oil supplementation in the parenteral nutrition of critically ill medical patients: a randomised controlled trial. | Friesecke S. et al. | P: ICU adult patients with sepsis<br><br>I: medium-chain triglycerides and long-chain triglycerides supplemented with fish oil<br><br>C: medium-chain triglycerides and long-chain triglycerides the same MCT/LCT emulsion supplemented with fish oil<br><br>O: Interleukin 6 (IL-6), monocyte HLA-DR ,infections, duration of mechanical ventilation, length of ICU stay, 28-day mortality |
| 21 | Comparative study on influence of enteral and parenteral nutrition on organ function in critically ill patients. | Xiang XJ. et al. | P: ICU adult patients<br><br>I: EN<br><br>C:PN<br><br>O:Mortality, ICU stay |
| 22 | Influence of n-3 polyunsaturated fatty acids enriched lipid emulsions on nosocomial infections and clinical outcomes in critically ill patients: ICU lipids study. | Grau-Carmona T. et al. | P: ICU adult patients<br><br>I: TPN enriched<br><br>C: TPN standard<br><br>O:nososcomial infections |
| 23 | Intravenous n-3 fatty acids in the critically ill. | Mayer K. et al. | Systematic review |
| 24 | Intravenous fish oil in adult intensive care unit patients. | Heller AR. et al. | Narrative review |
| 25 | The effect of L-alanyl-L-glutamine dipeptide supplemented total parenteral nutrition on infectious morbidity and insulin sensitivity in critically ill patients. | Grau T. et al. | P: ICU adult patients<br><br>I: TPN supplemented alanine-glutamine dipeptides |

| | | | |
|---|---|---|---|
| | | | C: TPN isonitrogenous and isocaloric<br><br>O: UTI, nosocomial infections, mortality |
| 26 | Influence of polymeric enteral nutrition supplemented with different doses of glutamine on gut permeability in critically ill patients. | Velasco N. et al. | P: ICU adult patients with intestinal disorders<br><br>I: EN supplemented with glutamine<br><br>C: EN standard<br><br>O:gut permeability/function |
| 27 | Zinc supplementation in critically ill patients: a key pharmaconutrient? | Heyland DK. et al. | Systematic review and meta-analysis |
| 28 | Randomised trial of glutamine, selenium, or both, to supplement parenteral nutrition for critically ill patients. | Andrews PJ. et al. | P: ICU adult patients with gastrointestinal failure<br><br>I:PN enriched with glutamine or selenium or both<br><br>C:PN standard<br><br>O:nosocomial infections, hospital stay, days of antibiotic use |
| 29 | Parenteral supplementation with EPA/DHA omega-3 fatty acids improves recovery prognosis in critically ill patients. | Lira Marcial E. et al. | In Spanish |
| 30 | Effects of early enteral arginine supplementation on resuscitation of severe burn patients. | Yan H. et al. | P: ICU patients with major burn TBSA >50%<br><br>I:1$^{st}$ group =EN enriched with 400mg/kg/day of L-arginine<br><br>2$^{nd}$ group= EN enriched with 200mg/kg/day of L-Arginine<br><br>C: EN standard<br><br>O:Mean arterial blood pressure, lactic acid concentration |
| 31 | Effects of lipid emulsions in parenteral nutrition of esophageal cancer surgical patients receiving enteral nutrition: a comparative analysis. | Wang WP. et al. | P: Patients with rejectable esophageal carcinoma in ICU and non-ICU department<br><br>I: EN+PN enriched with olive oil<br><br>C: EN+PN<br><br>O:Infections,length of hospitalization |

| | | | |
|---|---|---|---|
| 32 | Tight Blood-Glucose Control without Early Parenteral Nutrition in the ICU. | Gunst J. et al. | P: ICU adult patients<br>I: PN and liberal glucose control<br>C:PN and tight glucose control<br>O:Mortality, glucose levels |
| 33 | Uptake of dietary amino acids into arterial blood during continuous enteral feeding in critically ill patients and healthy subjects. | Liebau F. et al. | P: ICU adult patients and healthy<br>I: EN enriched with iv amino acids in ICU patients<br>C: EN enriched with amino acids iv in healthy people<br>O: essential amino acid concentrations and 13C-phenylalanine levels |
| 34 | Safety and metabolic tolerance of a concentrated long-chain triglyceride lipid emulsion in critically ill septic and trauma patients. | García-de-Lorenzo A. et al. | P: ICU adult patients after surgery or trauma<br>I: TPN enriched 30% LCT<br>C: TPN enriched 20% LCT<br>O: Biochemical markers (prealbumin, free cholesterol, triglycerides) |
| 35 | Applied studies of structured triglycerides for parenteral nutrition in severe hemorrhagic shock patients after resuscitation. | Su MS. et al. | P: ICU adult patients after severe blood loss >3000ml<br>I:PN with structured triglycerides<br>C:PN with MCT/LCT<br>O:serum triglycerides levels, prealbumin, transferrin |
| 36 | A prospective randomized study of preoperative nutritional supplementation in patients awaiting elective orthotopic liver transplantation. | Le Cornu KA. et al. | P: patients undergoing liver transplantation<br>I: EN added to usual diet<br>C: usual diet<br>O: nutritional status, improved mid-arm circumference, mid-arm muscle circumference, and grip strength |
| 37 | A prospective, randomized, controlled study of ω-3 fish oil fat emulsion-based parenteral nutrition for patients following surgical resection of gastric tumors. | Wei Z. et al. | P: Patients after gastric tumor surgery<br>I: TPN with ω-3 fish oil<br>C: TPN with soybean oil |

| | | | |
|---|---|---|---|
| | | | O: Nutritional status, infections |
| 38 | Beta-hydroxy-beta-methylbutyrate supplementation in critically ill trauma patients. | Kuhls DA. et al. | P: ICU trauma patients<br><br>I: 1ST EN with β-hydroxy-β-methylbutyrate<br><br>2nd EN with arginine 3rd EN with glutamine<br><br>C: EN standard<br><br>O:Nitrogen balance and muscle proteolysis |
| 39 | Parenteral nutrition effects of Omega-3 fatty acids on C-reactive protein, high-density lipoprotein, lymphocyte characteristics and the treatment of critically ill patients. | Ni C. et al. | P: Critically ill cancer patients<br><br>I: EN+PN+Ω3 fatty acids<br><br>C: EN+PN standard regimes<br><br>O: nutritional status, inflammatory markers, ICU stay, ICU mortality |
| 40 | Pilot study on the effect of parenteral vitamin E on ischemia and reperfusion induced liver injury: a double blind, randomized, placebo-controlled trial. | Bartels M. et al. | P: Patients a day before elective liver tumor resection<br><br>I: Standard diet and 3 infusions iv of vitamin E<br><br>C: Standard diet and 3 infusions iv of placebo<br><br>O: ALT levels, length of ICU stay |
| 41 | Enteral versus parenteral early nutrition in ventilated adults with shock: a randomised, controlled, multicenter, open-label, parallel-group study (NUTRIREA-2). | Reignier J. et al. | P: ICU patients<br><br>I: EN<br><br>C:PN<br><br>O:Mortality,length of stay in ICU |
| 42 | Enteral or parenteral nutrition in traumatic brain injury: a prospective randomised trial. | Justo Meirelles CM. et al. | P: Patients with traumatic brain injury<br><br>I: EN<br><br>C:PN |

| | | | |
|---|---|---|---|
| | | | O:Mortality |
| 43 | Effect of enteral versus parenteral nutrition on outcome of medical patients requiring mechanical ventilation. | Altintas ND. et al. | P: ICU patients<br><br>I: EN<br><br>C:PN<br><br>O:Mortality,length of ICU stay |
| 44 | Phase 3 Pilot Randomized Controlled Trial Comparing Early Trophic Enteral Nutrition With ""No Enteral Nutrition"" in Mechanically Ventilated Patients With Septic Shock. | Patel JJ. et al. | P: ICU ventilated patients with septic shock<br><br>I: Early trophic EN<br><br>C: Nil per os<br><br>O:Mortality, infections |
| 45 | Effect of late versus early initiation of parenteral nutrition on weight deterioration during PICU stay: Secondary analysis of the PEPaNIC randomised controlled trial. | van Puffelen E. et al. | P: PICU critically ill patients<br><br>I: EPN<br><br>C: LPN<br><br>O:Mortality, hospitalization time |
| 46 | A multicenter, randomised controlled trial comparing the clinical effectiveness and cost-effectiveness of early nutritional support via the parenteral versus or the enteral route in critically ill patients (CALORIES). | Harvey SE. et al. | P: Critically ill patients in ICU<br><br>I: EN<br><br>C:PN<br><br>O:Cost effectiveness |
| 47 | Early metabolic support for critically ill trauma patients: A prospective randomized controlled trial. | Stolarski AE. et al. | P: Patients after abdominal surgery<br><br>I: High protein supplementation<br><br>C: Usual care<br><br>O: Total calories delivery |
| 48 | Effect of an enteral diet supplemented with a specific blend of amino acid on | Mansoor O. et al. | P: Critically ill patients in ICU<br><br>I: EN supplemented with amino acids |

| | | | |
|---|---|---|---|
| | plasma and muscle protein synthesis in ICU patients. | | C: EN standard<br><br>O: Mortality, muscle protein synthesis |
| 49 | A randomized controlled trial of enteral versus parenteral feeding in patients with predicted severe acute pancreatitis shows a significant reduction in mortality and in infected pancreatic complications with total enteral nutrition. | Petrov MS. et al. | P: Patients with severe acute pancreatitis<br><br>I: EN<br><br>C:PN<br><br>O: Mortality, pancreatitis complications |
| 50 | The effect of L-alanyl-L-glutamine dipeptide supplemented total parenteral nutrition on infectious morbidity and insulin sensitivity in critically ill patients. | Grau T. et al. | P: Critically ill patients<br><br>I: TPN supplemented L-alanyl-L-glutamine dipeptide<br><br>C: TPN standard<br><br>O: Mortality, insulin resistance |
| 51 | Structured lipid emulsion as nutritional therapy for the elderly patients with severe sepsis. | Chen J. et al. | P: Patients with sepsis<br><br>I: EN+PN of structured lipid emulsion<br><br>C: EN+PN of mixed medium and long-chain fat<br><br>O: albumin, prealbumin, cholesterol, and triglyceride levels, hospitalization days |
| 52 | The influence of parenteral glutamine supplementation on glucose homeostasis in critically ill polytrauma patients--A randomized-controlled clinical study. | Grintescu IM. et al. | P: Critically ill polytrauma patients<br><br>I: PN of glutamine<br><br>C: standard care<br><br>O: Glucose levels, mortality |
| 53 | Randomised trial of glutamine and selenium supplemented parenteral nutrition for critically ill patients. Protocol Version 9, 19 February 2007 known as SIGNET (Scottish Intensive care Glutamine or selenium Evaluative Trial). | Andrews PJ. et al. | P: Critically ill adult patients in ICU<br><br>I: PN supplemented with glutamine and selenium<br><br>C:PN standard<br><br>O: Mortality, length of stay ICU |
| 54 | Omega-3 fatty acids-supplemented parenteral nutrition decreases | Wang X. et al. | P: Critically ill patients with severe acute pancreatitis |

| | | | |
|---|---|---|---|
| | hyperinflammatory response and attenuates systemic disease sequelae in severe acute pancreatitis: a randomized and controlled study. | | I: Omega-3 fatty acids-supplemented parenteral nutrition<br><br>C:PN standard<br><br>O: infection morbidity, mortality, intensive care unit time, and length of hospital stay |
| 55 | NutriSup-PPN: A pilot randomized control trial of oral nutritional supplementation (ONS) and peripheral parenteral nutrition (PPN) in canadian, malnourished, hospitalized patients. | Mrkobrada M. et al. | P: Malnourished adult patients in non-ICU department<br><br>I: Oral nutrition and LPN in 30 day<br><br>C: Oral nutrition and PN<br><br>O: nutritional status, length of stay in hospital, mortality |
| 56 | Administration of Free Amino Acids Improves Exogenous Amino Acid Availability when Compared with Intact Protein in Critically Ill Patients: A Randomized Controlled Study. | van Gassel RJ. et al. | P: ICU adult patients under mechanical ventilation<br><br>I: EN with 20 g intrinsically L-[1-13C]-phenylalanine-labeled milk protein<br><br>C: EN with equivalent number of amino acids labeled with free L-[1-13C]-phenylalanine<br><br>O: Protein digestion and amino acid absorption kinetics and whole-body protein net balance were assessed throughout a 6-h period |
| 57 | Impact of n-3 fatty acid supplemented parenteral nutrition on hemostasis patterns after major abdominal surgery. | Heller AR. et al. | P: Patients after major abdominal surgery<br><br>I: n-3 fatty acid supplemented PN<br><br>C:PN standard<br><br>O: Nutritional status, mortality |
| 58 | Clinical trial of treating stress-induced hyperglycemia patients with sepsis by supplementing QI, nourishing yin, and promoting blood flow. | Gao ZL. et al. | P: stress-induced hyperglycemia patients with sepsis<br><br>I: supplementing QI, nourishing yin<br><br>C: serum levels of CRP, TNF-alpha, IL-1 and IL-6 |
| 59 | Intravenous glutamine supplementation to head trauma patients leaves cerebral glutamate concentration unaffected. | Berg A. et al. | P: Head trauma patients<br><br>I:PN with glutamine<br><br>C:PN standard<br><br>O:Mortality |

| | | | |
|---|---|---|---|
| 60 | Superiority of a fish oil-enriched emulsion to medium-chain triacylglycerols/long-chain triacylglycerols in gastrointestinal surgery patients: a randomized clinical trial. | Wang J. et al. | P: Patients after GI surgery<br>I: TPN with Ω-3<br>C: TPN with MCT/LCT<br>O:Infections, mortality |
| 61 | Effectiveness and safety of two lipid emulsions for parenteral nutrition in postsurgical critically ill patients: Clinoleic® versus SMOFlipid®. | Martínez-Lozano Aranaga F | P: postsurgical critically ill patients<br>I: Clinoleic®<br>C: SMOFlipid®<br>O: Infections |
| 62 | Effects of Early Enteral Glutamine Supplementation on Intestinal Permeability in Critically Ill Patients. | Shariatpanahi ZV. et al. | P: Critically ill patients<br>I: EN with glutamine<br>C: EN standard<br>O: Intestinal tolerance |
| 63 | Preoperative non-selective administration of nutritional supplements to patients undergoing elective colorectal resection - standard of perioperative care? | Tesař M. et al. | P: Patients undergoing elective colorectal resection<br>I: ONS<br>C: Standard care<br>O: Postoperative biomarkers, length of hospital stay |
| 64 | Short-term infusion of a fish oil-based lipid emulsion modulates fatty acid status, but not immune function or (anti)oxidant balance: a randomized cross-over study. | Versleijen MW. et al. | P: Critically ill patients with sepsis or trauma<br>I:PN with fish oil<br>C:PN with soybean oil<br>O: fatty acid incorporation, immune functions and (anti)oxidant balance |
| 65 | Adapted ERAS Pathway Versus Standard Care in Patients Undergoing Emergency Surgery for Perforation Peritonitis-a Randomized Controlled Trial. | Pranavi AR. et al. | P: Patients after emergency surgery due to peritonitis<br>I: EEN<br>C: Standard care |

| | | | |
|---|---|---|---|
| | | | O: Mortality |
| 66 | Clinical nursing application of parenteral nutrition combined with enteral nutrition support in neurosurgery. | Huang M. et al. | P: patients in neurosurgery department after surgery<br><br>I: EN+PN<br><br>C:PN<br><br>O: Survival |
| 67 | Impact of supplemental parenteral nutrition early during critical illness on invasive fungal infections: a secondary analysis of the EPaNIC randomized controlled trial. | De Vlieger G. et al. | P: Critically ill ICU patients<br><br>I: EPN<br><br>C: LPN<br><br>O: Effect on fungal nosocomial infections |
| 68 | Influence of Early versus Late supplemental ParenteraL Nutrition on long-term quality of life in ICU patients after gastrointestinal oncological surgery (hELPLiNe): study protocol for a randomized controlled trial. | Piwowarczyk P. et al. | P: ICU patients after major tumor resection<br><br>I: EN+EPN<br><br>C: EN+LPN<br><br>O: Mortality, nutritional status |
| 69 | Glutamine and antioxidants in the critically ill patient: a post hoc analysis of a large-scale randomized trial. | Heyland DK. et al. | P: Critically ill patients in ICU<br><br>I1: EN+PN of glutamine<br><br>12: EN+PN of antioxidants<br><br>I3: EN+PN of glutamine and antioxidants<br><br>C: Standard EN +PN |
| 70 | Regulation of omega-3 fish oil emulsion on the SIRS during the initial stage of severe acute pancreatitis. | Xiong J. et al. | P: Patients with severe acute pancreatitis<br><br>I: iv fluids and iv Ω-3 fatty acids<br><br>C: iv fluids<br><br>O: Mortality |

| | | | |
|---|---|---|---|
| 71 | A supplemental intravenous amino acid infusion sustains a positive protein balance for 24 hours in critically ill patients. | Sundström Rehal M. et al. | P: Critically ill patients<br><br>I: iv amino acids and measurement of serum protein levels at 3h and 24h of infusion<br><br>O: Protein concentration |
| 72 | The effects of intravenous, enteral and combined administration of glutamine on malnutrition in sepsis: a randomized clinical trial. | Koksal GM. et al. | P: ICU patients with sepsis<br><br>I1: iv glutamine<br><br>12: EN enriched glutamine<br><br>I3: EN enriched glutamine and iv supplementation<br><br>C: EN standard<br><br>O: nitrogen balance, creatinine levels |
| 73 | Reducing In-Hospital and 60-Day Mortality in Critically Ill Patients after Surgery with Strict Nutritional Supplementation: A Prospective, Single-Labeled, Randomized Controlled Trial. | Kim EY. et al. | P: Critically ill adult patients<br><br>I: EN enriched with proteins<br><br>C: EN standard<br><br>O: Mortality, length of hospitalization |
| 74 | Enteral Omega-3 Fatty Acid, γ-Linolenic Acid, and Antioxidant Supplementation in Acute Lung Injury. | Rice W. et al. | P: Critically ill patients with lung injury<br><br>I: EN with Ω3 fatty acids or antioxidants or γ-linolenic acid<br><br>C: EN standard<br><br>O: Mortality |
| 75 | Early use os supplemental parenteral nutrition in critically ill patients: Results of an international multicenter observational study, | Koutsogiannis,Alberta C. et al. | Observational study |

| | | | |
|---|---|---|---|
| 76 | Factors associated with the need of parenteral nutrition in critically ill patients after initiation of enteral nutrition therapy. | Lopez C. et al. | Observational study |
| 77 | The effect of continuous enteral nutrition on nutrition indices, compared to the intermittent and combination enteral nutrition in traumatic brain injury patients. | Mazaherpur S. et al. | P: Critically ill patients in ICU with brain injury<br>I1: Intermittent EN<br>I2: Continuous EN<br>I3: Combination of them<br>O: Mortality |
| 78 | Comparison of enteral nutrition with combined enteral and parenteral nutrition in post-pancreaticoduodenectomy patients: a pilot study. | Shigeyuki N. et al. | P: Patients after elective pancreaticoduodenectomy in surgery department due to carcinoma<br>I: EN+PN<br>C: EN<br>O:Mortality |
| 79 | Effect of Early vs Late Supplemental Parenteral Nutrition in Patients Undergoing Abdominal Surgery A Randomized Clinical Trial. | Xuejin G. et al. | P: Patients after elective abdominal surgery<br>I: EN+SPN<br>C: EN+LPN<br>O:Mortality |
| 80 | Energy requirements and the use of predictive equations versus indirect calorimetry in critically ill patients | Wichansawakun S. et al. | Narrative review |

**Supplementary Table S6**: AMSTAR 2.0 tool for the assessment of the included systematic reviews

| | Items | | | | | | | | | | | | | | | | | |
|---|---|---|---|---|---|---|---|---|---|---|---|---|---|---|---|---|---|---|
| **Study** | **Q1** | **Q2** | **Q3** | **Q4** | **Q5** | **Q6** | **Q7** | **Q8** | **Q9 RCT** | **Q9 NRSI** | **Q10** | **Q11 RCT** | **Q11 NRSI** | **Q12** | **Q13** | **Q14** | **Q15** | **Q16** | **Overall Quality** |
| Alsharif et al., 2020 | Y | Y | N | PY | Y | Y | Y | Y | N | NA | N | Y | NA | Y | Y | Y | N | Y | Critically Low |
| Dhaliwal et al., 2004 | Y | Y | N | N | N | N | N | N | N | NA | N | N | NA | NA | NA | NA | NA | N | Critically Low |
| Hill et al., 2022 | Y | Y | N | PY | Y | N | Y | PY | Y | NA | N | Y | NA | Y | Y | Y | N | Y | Low |
| Lewis et al., 2018 | Y | Y | N | Y | Y | Y | Y | PY | Y | NA | Y | Y | NA | Y | Y | Y | Y | Y | High |
| Shi et al., 2018 | Y | N | N | PY | N | N | PY | PY | Y | NA | N | Y | NA | Y | Y | Y | N | Y | Low |

N: No; NA: Not Applicable; NI: No Information; NRSI: Non-Randomized Intervention Study; PY: Partial Yes; RCT: Randomized Controlled Trial; Y: Yes

**Supplementary Table S7:** Assessment of certainty of evidence according to GRADE

| **Supplementary Table S7:** Assessment of certainty of evidence according to GRADE **Certainty assessment** | | | | | | | **No of patients** | | **Effect** | | **Certainty** | **Importance** |
|---|---|---|---|---|---|---|---|---|---|---|---|---|
| **No of studies** | **Study design** | **Risk of bias** | **Inconsistency** | **Indirectness** | **Imprecision** | **Other considerations** | **EN+PN** | **EN** | **Relative (95%CI)** | **Absolute (95%CI)** | | |
| Overall mortality | | | | | | | | | | | | |
| 12 | RCTs | serious[a] | Not serious | Serious [b] | Not serious | - | 2754 | 2797 | RR 0.89 (0.72; 1.11) | - | + - + - Low/Very low | - |
| ICU mortality in 30 days | | | | | | | | | | | | |
| 2 | RCTs | serious[a] | Not serious | Serious [b] | Not serious | - | 183 | 183 | RR 0.66(0.42; 1.01) | - | + - + - Low/ Very low | - |
| Overall hospitalization days | | | | | | | | | | | | |
| 10 | RCTs | serious[a] | Not serious | Serious [b] | Not serious | - | 2729 | 2765 | - | MD 0.48 (-2.40; 3.36) | + - + - Low/ Very low | - |
| LOS in ICU in days | | | | | | | | | | | | |
| 11 | RCTs | serious[a] | Not serious | Serious [b] | Not serious | - | 2741 | 2772 | - | MD -0.19 (-1.19; 0.80) | + - + - Low/ Very low | - |
| Duration of mechanical ventilation | | | | | | | | | | | | |
| 11 | RCTs | serious[a] | Not serious | Serious [b] | Not serious | - | 2741 | 2772 | - | MD -0.52 (-1.38; 0.33) | + - + - Low/ Very low | - |

**CI:** confidence interval; **MD**: mean difference; **RR**: risk ratio; Explanations: a. Lack of allocation concealment and absence of pre-specified protocol. b. Patients with a variety of critical conditions.

**Supplementary Figure S1**: PRISMA flow diagram for the selection process of the systematic reviews.

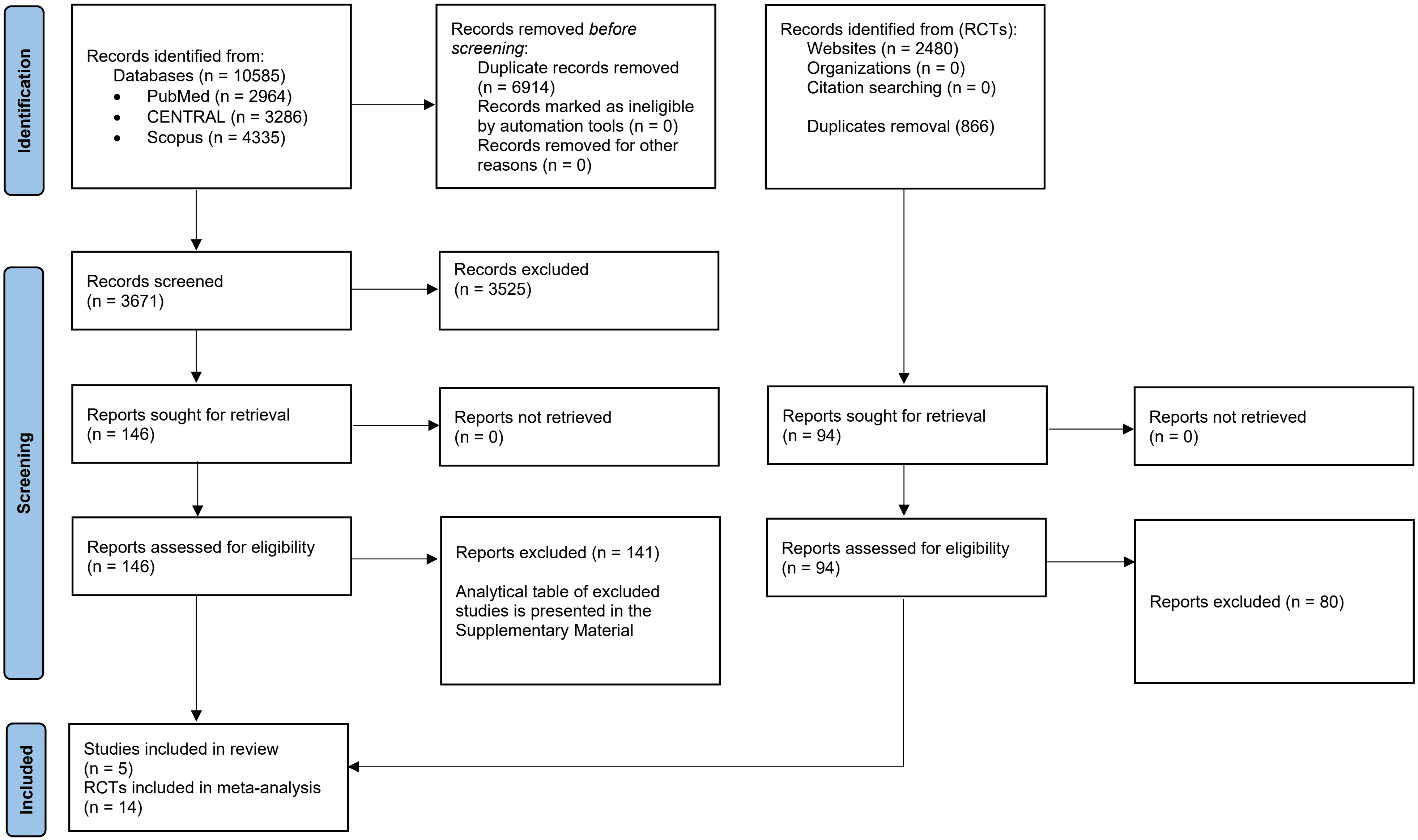

**Supplementary Figure S2**: Corrected covered area and corrected covered area after adjusting by structural zeros, respectively

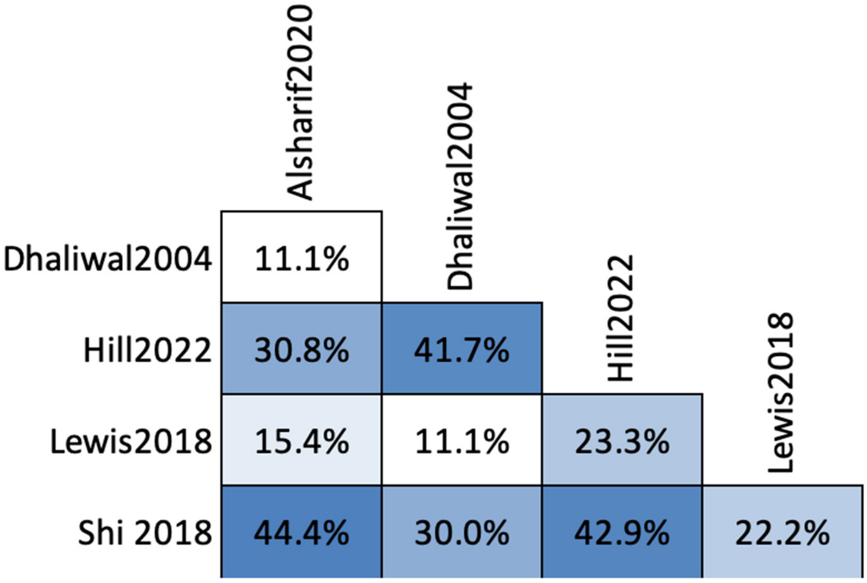


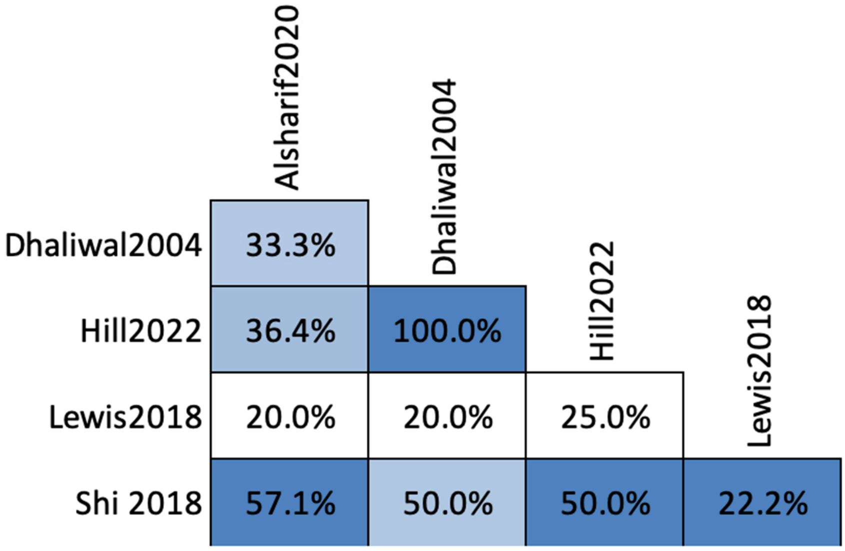

**Supplementary Figure S3**: Effect of EN+PN compared to EN alone on overall mortality

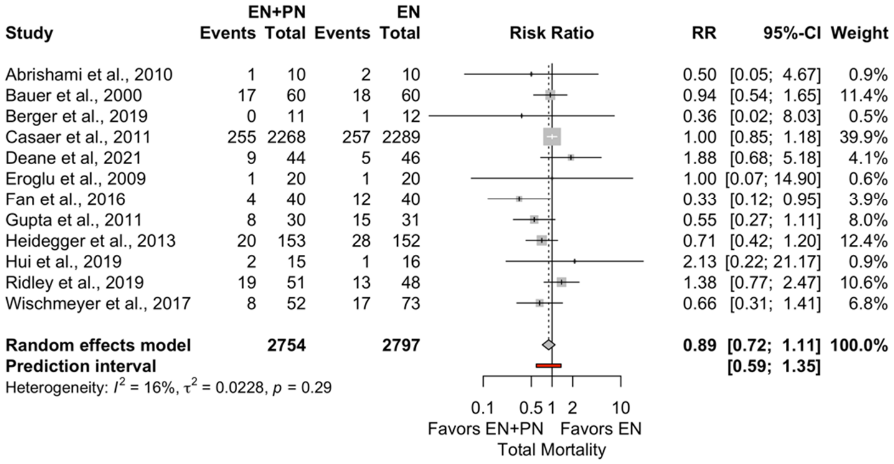

**Supplementary Figure S4**: Effect of EN+PN compared to EN alone on mortality in the ICU

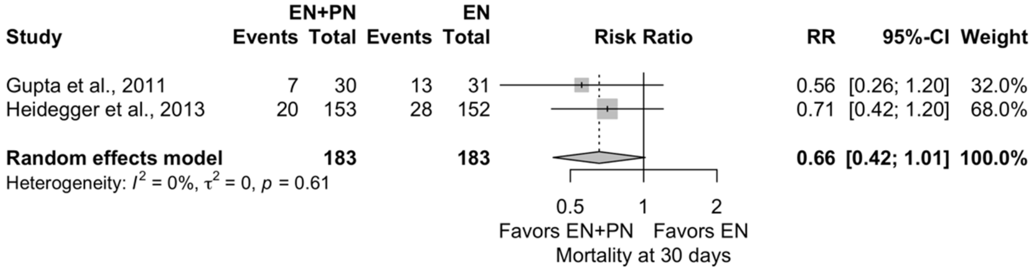

**Supplementary Figure S5**: Effect of EN+PN compared to EN alone on hospitalization

| Study | EN+PN Total | EN+PN Mean | EN+PN SD | EN Total | EN Mean | EN SD | Mean Difference | MD | 95%-CI | Weight |
|---|---|---|---|---|---|---|---|---|---|---|
| Bauer et al., 2000 | 60 | 31.20 | 18.5000 | 60 | 33.70 | 27.7000 | | -2.50 | [-10.93; 5.93] | 7.3% |
| Berger et al., 2018 | 11 | 43.63 | 22.9000 | 12 | 43.61 | 28.5100 | | 0.02 | [-21.04; 21.08] | 1.7% |
| Casaer et al., 2011 | 2312 | 18.10 | 14.8400 | 2328 | 16.80 | 13.3500 | | 1.30 | [ 0.49; 2.11] | 19.4% |
| Deane et al., 2021 | 44 | 18.94 | 11.5000 | 46 | 22.48 | 19.1300 | | -3.54 | [-10.03; 2.95] | 9.8% |
| Gupta et al., 2011 (non survivors) | 9 | 12.89 | 12.6300 | 14 | 10.93 | 14.6200 | | 1.96 | [ -9.30; 13.22] | 4.9% |
| Gupta et al., 2011 (survivors) | 22 | 21.50 | 13.4900 | 16 | 26.63 | 18.2200 | | -5.13 | [-15.69; 5.43] | 5.4% |
| Heidegger et al., 2013 | 153 | 31.00 | 23.0000 | 152 | 32.00 | 23.0000 | | -1.00 | [ -6.16; 4.16] | 12.1% |
| Hui et al., 2019 | 15 | 29.10 | 1.9000 | 16 | 22.30 | 1.2000 | | 6.80 | [ 5.67; 7.93] | 19.1% |
| Ridley et al., 2018 | 51 | 22.00 | 21.0000 | 48 | 23.00 | 17.0000 | | -1.00 | [ -8.51; 6.51] | 8.4% |
| Wischmeyer et al., 2017 | 52 | 25.34 | 13.1100 | 73 | 26.65 | 16.8700 | | -1.31 | [ -6.57; 3.95] | 11.9% |
| **Random effects model** | **2729** | | | **2765** | | | | **0.48** | **[ -2.40; 3.36]** | **100.0%** |
| **Prediction interval** | | | | | | | | | **[ -7.88; 8.83]** | |

Heterogeneity: $I^2$ = 88%, $\tau^2$ = 10.9670, $p$ < 0.01

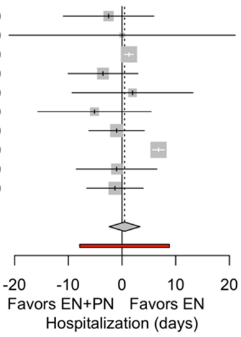

**Supplementary Figure S6**: Effect of EN+PN compared to EN alone on ICU length of stay

| Study | EN+PN Total | EN+PN Mean | EN+PN SD | EN Total | EN Mean | EN SD | MD | 95%-CI | Weight |
|---|---|---|---|---|---|---|---|---|---|
| Bauer et al., 2000 | 60 | 16.90 | 11.8000 | 60 | 17.30 | 12.8000 | -0.40 | [ -4.81; 4.01] | 4.3% |
| Berger et al., 2018 | 11 | 14.34 | 5.7700 | 12 | 14.08 | 14.5100 | 0.26 | [ -8.63; 9.15] | 1.2% |
| Casaer et al., 2011 | 2312 | 5.05 | 5.1900 | 2328 | 4.05 | 3.7100 | 1.00 | [ 0.74; 1.26] | 26.6% |
| Eroglu et al., 2009 | 20 | 14.00 | 2.0000 | 20 | 15.00 | 2.0000 | -1.00 | [ -2.24; 0.24] | 19.1% |
| Fan et al., 2016 | 40 | 27.61 | 7.5400 | 40 | 31.42 | 5.9300 | -3.81 | [ -6.78; -0.84] | 7.9% |
| Gupta et al., 2011 (non survivors) | 7 | 12.89 | 12.6300 | 13 | 10.86 | 14.5900 | 2.03 | [-10.24; 14.30] | 0.6% |
| Gupta et al., 2011 (survivors) | 24 | 15.96 | 8.5500 | 17 | 15.88 | 8.6100 | 0.08 | [ -5.25; 5.41] | 3.1% |
| Heidegger et al., 2013 | 153 | 13.00 | 10.0000 | 152 | 13.00 | 11.0000 | 0.00 | [ -2.36; 2.36] | 10.7% |
| Luo et al., 2008 | 11 | 7.60 | 2.3200 | 9 | 6.90 | 2.7000 | 0.70 | [ -1.53; 2.93] | 11.4% |
| Ridley et al., 2018 | 51 | 11.00 | 9.1500 | 48 | 11.35 | 8.4100 | -0.35 | [ -3.81; 3.11] | 6.3% |
| Wischmeyer et al., 2017 | 52 | 12.84 | 7.5500 | 73 | 13.16 | 8.0200 | -0.32 | [ -3.08; 2.44] | 8.8% |
| **Random effects model** | **2741** | | | **2772** | | | **-0.19** | **[ -1.19; 0.80]** | **100.0%** |
| **Prediction interval** | | | | | | | | **[ -2.68; 2.29]** | |

Heterogeneity: $I^2 = 53\%$, $\tau^2 = 0.9485$, $p = 0.02$

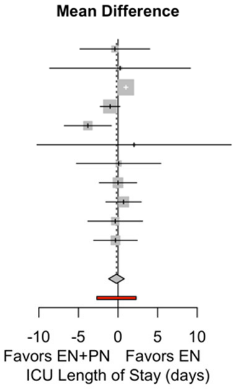

**Supplementary Figure S7**: Effect of EN+PN compared to EN alone on mechanical ventilation support days

| Study | EN+PN Total | EN+PN Mean | EN+PN SD | EN Total | EN Mean | EN SD | MD | 95%-CI | Weight |
|---|---|---|---|---|---|---|---|---|---|
| Bauer et al., 2000 | 60 | 11.00 | 9.0000 | 60 | 10.00 | 8.0000 | 1.00 | [ -2.05; 4.05] | 6.3% |
| Berger et al., 2018 | 11 | 9.93 | 9.1600 | 12 | 8.32 | 8.6400 | 1.61 | [ -5.68; 8.90] | 1.3% |
| Casaer et al., 2011 | 2312 | 2.70 | 2.9700 | 2328 | 2.70 | 2.9700 | 0.00 | [ -0.17; 0.17] | 30.6% |
| Eroglu et al., 2009 | 20 | 8.00 | 3.0000 | 20 | 9.00 | 3.0000 | -1.00 | [ -2.86; 0.86] | 12.6% |
| Fan et al., 2016 | 40 | 8.39 | 4.6700 | 40 | 12.56 | 6.1200 | -4.17 | [ -6.56; -1.78] | 9.1% |
| Gupta et al., 2011 (non survivors) | 7 | 11.78 | 10.6300 | 13 | 10.71 | 14.5500 | 1.07 | [-10.09; 12.23] | 0.6% |
| Gupta et al., 2011 (survivors) | 24 | 13.86 | 7.5700 | 17 | 11.75 | 6.4700 | 2.11 | [ -2.21; 6.43] | 3.5% |
| Heidegger et al., 2013 | 153 | 6.38 | 6.7900 | 152 | 6.92 | 6.6700 | -0.54 | [ -2.05; 0.97] | 15.8% |
| Luo et al., 2008 | 11 | 5.00 | 3.3200 | 9 | 6.00 | 3.0000 | -1.00 | [ -3.77; 1.77] | 7.3% |
| Ridley et al., 2018 | 51 | 10.35 | 6.8700 | 48 | 10.48 | 9.9400 | -0.13 | [ -3.52; 3.26] | 5.3% |
| Wischmeyer et al., 2017 | 52 | 8.27 | 7.7800 | 73 | 8.48 | 7.1800 | -0.21 | [ -2.89; 2.47] | 7.7% |
| **Random effects model** | **2741** | | | **2772** | | | **-0.52** | **[ -1.38; 0.33]** | **100.0%** |
| **Prediction interval** | | | | | | | | **[ -2.56; 1.51]** | |

Heterogeneity: $I^2 = 35\%$, $\tau^2 = 0.6172$, $p = 0.12$

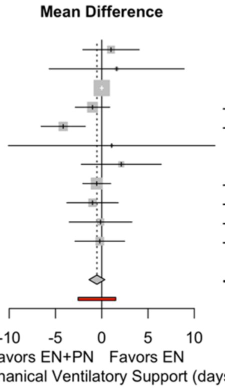

**Supplementary Figure S8**: Effect of EN+PN compared to EN alone on respiratory infections

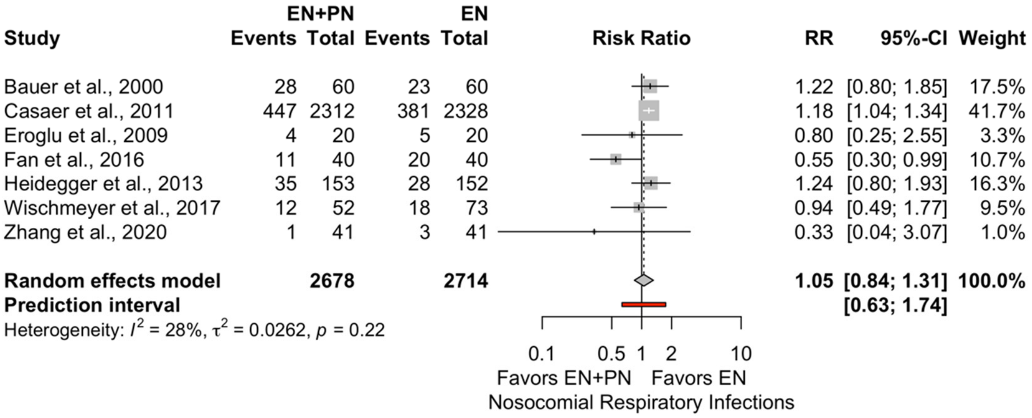

**Supplementary Figure S9**: Effect of EN+PN compared to EN alone on bloodstream infections

| Study | EN+PN Events | EN+PN Total | EN Events | EN Total | RR | 95%-CI | Weight |
|---|---|---|---|---|---|---|---|
| Casaer et al., 2011 | 174 | 2312 | 142 | 2328 | 1.23 | [1.00; 1.53] | 91.0% |
| Eroglu et al., 2009 | 2 | 20 | 3 | 20 | 0.67 | [0.12; 3.57] | 1.5% |
| Fan et al., 2016 | 10 | 40 | 3 | 40 | 3.33 | [0.99; 11.22] | 2.8% |
| Heidegger et al., 2013 | 10 | 153 | 6 | 152 | 1.66 | [0.62; 4.44] | 4.3% |
| Wischmeyer et al., 2017 | 0 | 52 | 1 | 73 | 0.47 | [0.02; 11.23] | 0.4% |
| **Random effects model** | | **2577** | | **2613** | **1.27** | **[1.03; 1.56]** | **100.0%** |
| **Prediction interval** | | | | | | **[0.91; 1.77]** | |

Heterogeneity: $I^2 = 0\%$, $\tau^2 = 0$, $p = 0.44$

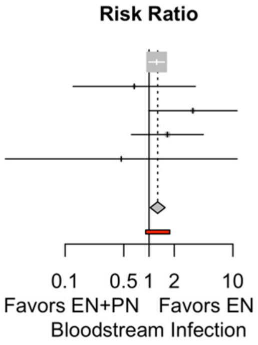

**Supplementary Figure S10**: Effect of EN+PN compared to EN alone on vomiting

| Study | EN+PN Events | EN+PN Total | EN Events | EN Total | RR | 95%-CI | Weight |
|---|---|---|---|---|---|---|---|
| Ridley et al., 2018 | 13 | 51 | 8 | 48 | 1.53 | [0.70; 3.36] | 94.2% |
| Zhang et al., 2020 | 0 | 41 | 1 | 41 | 0.33 | [0.01; 7.95] | 5.8% |
| **Random effects model** | | **92** | | **89** | **1.40** | **[0.65; 3.01]** | **100.0%** |

Heterogeneity: $I^2 = 0\%$, $\tau^2 = 0$, $p = 0.36$

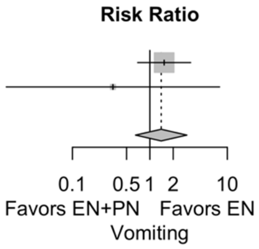

**Supplementary Figure S11**: Effect of EN+PN compared to EN alone on diarrhea

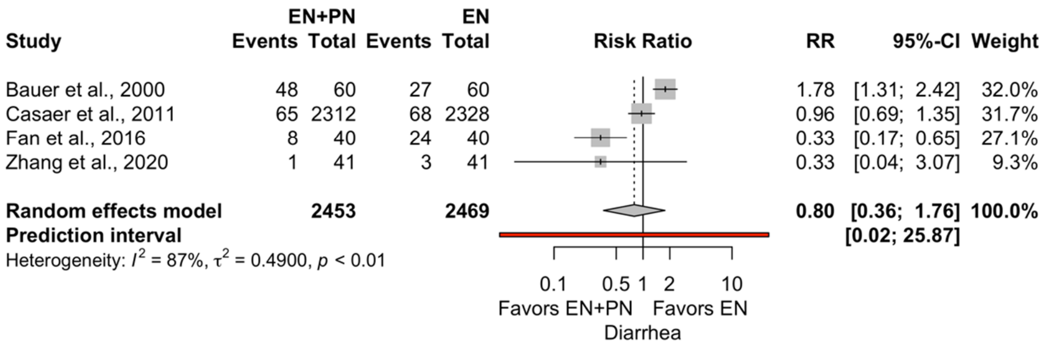

**Supplementary Figure S12**: Effect of EN+PN compared to EN alone on albumin (g/l)

| Study | EN+PN Total | EN+PN Mean | EN+PN SD | EN Total | EN Mean | EN SD | MD | 95%-CI | Weight |
|---|---|---|---|---|---|---|---|---|---|
| Bauer et al., 2000 | 57 | 20.70 | 5.6000 | 56 | 20.40 | 5.9000 | 0.30 | [ -1.82; 2.42] | 25.6% |
| Fan et al., 2016 | 40 | 39.92 | 8.1300 | 40 | 38.57 | 7.6900 | 1.35 | [ -2.12; 4.82] | 21.6% |
| Hui et al., 2019 | 15 | 36.00 | 3.4000 | 16 | 41.00 | 0.6000 | -5.00 | [ -6.75; -3.25] | 26.5% |
| Zhang et al., 2020 | 41 | 36.87 | 4.7800 | 41 | 34.91 | 3.4800 | 1.96 | [ 0.15; 3.77] | 26.3% |
| **Random effects model** | **153** | | | **153** | | | **-0.44** | **[ -3.68; 2.80]** | **100.0%** |
| **Prediction interval** | | | | | | | | **[-15.50; 14.62]** | |

Heterogeneity: $I^2 = 91\%$, $\tau^2 = 9.5188$, $p < 0.01$

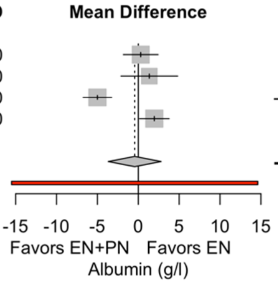

**Supplementary Figure S13**: Effect of EN+PN compared to EN alone on glucose (mg/dl)

| Study | EN+PN Total | EN+PN Mean | EN+PN SD | EN Total | EN Mean | EN SD | MD | 95%-CI | Weight |
|---|---|---|---|---|---|---|---|---|---|
| Bauer et al., 2000 | 57 | 129.00 | 39.0000 | 56 | 131.00 | 49.0000 | -2.00 | [-18.35; 14.35] | 61.5% |
| Berger et al., 2018 | 11 | 130.39 | 37.7400 | 12 | 129.42 | 43.7800 | 0.97 | [-32.36; 34.30] | 14.8% |
| Luo et al., 2008 | 9 | 143.00 | 18.0000 | 9 | 145.00 | 36.0000 | -2.00 | [-28.30; 24.30] | 23.8% |
| **Random effects model** | **77** | | | **77** | | | **-1.56** | **[-14.38; 11.25]** | **100.0%** |
| **Prediction interval** | | | | | | | | **[-84.64; 81.52]** | |

Heterogeneity: $I^2 = 0\%$, $\tau^2 = 0$, $p = 0.99$

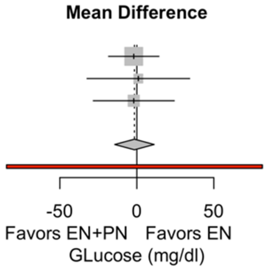

**Supplementary Figure S14**: Effect of EN+PN compared to EN alone on CRP (mg/l)

| Study | EN+PN Total | EN+PN Mean | EN+PN SD | EN Total | EN Mean | EN SD | Mean Difference | MD | 95%-CI | Weight |
|---|---|---|---|---|---|---|---|---|---|---|
| Bauer et al., 2000 | 57 | 113.80 | 78.7000 | 56 | 120.30 | 81.7000 | | -6.50 | [ -36.09; 23.09] | 31.2% |
| Casaer et al., 2011 | 2321 | 162.64 | 118.0900 | 2328 | 184.58 | 120.4600 | | -21.94 | [ -28.80; -15.08] | 44.0% |
| Ridley et al., 2018 | 32 | 158.34 | 86.1400 | 37 | 125.28 | 82.5200 | | 33.06 | [ -6.91; 73.03] | 24.8% |
| **Random effects model** | **2410** | | | **2421** | | | | **-3.46** | **[ -33.22; 26.30]** | **100.0%** |
| **Prediction interval** | | | | | | | | | **[-349.68; 342.76]** | |

Heterogeneity: $I^2 = 75\%$, $\tau^2 = 511.9102$, $p = 0.02$

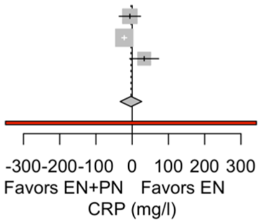

Supplement: Supplementary file 1 [file jcm-14-00991-s001.zip › jcm-3411363-supplementary.pdf]
